# Disentangling the heterogeneity of multiple sclerosis through identification of independent neuropathological dimensions

**DOI:** 10.1007/s00401-024-02742-w

**Published:** 2024-05-21

**Authors:** Alyse de Boer, Aletta M. R. van den Bosch, Nienke J. Mekkes, Nina L. Fransen, Ekaterina Dagkesamanskaia, Eric Hoekstra, Jörg Hamann, Joost Smolders, Inge Huitinga, Inge R. Holtman

**Affiliations:** 1 Section Molecular Neurobiology, Department of Biomedical Sciences, University Medical Center Groningen, University of Groningen, Groningen, The Netherlands; 2 Neuroimmunology Research Group, Netherlands Institute for Neuroscience, Amsterdam, The Netherlands; 3 Machine Learning Lab, Data Science Center in Health, University Medical Center Groningen, University of Groningen, Groningen, The Netherlands; 4 Department of Experimental Immunology, Amsterdam Institute for Immunology and Infectious Diseases, Amsterdam University Medical Center, Amsterdam, The Netherlands; 5 MS Center ErasMS, Departments of Neurology and Immunology, Erasmus MC, University Medical Center Rotterdam, Rotterdam, The Netherlands; 6 Swammerdam Institute for Life Sciences, Center for Neuroscience, University of Amsterdam, Amsterdam, The Netherlands; 7 The Netherlands Brain Bank, Netherlands Institute for Neuroscience, Amsterdam, The Netherlands

**Keywords:** Multiple sclerosis, Neuropathology, Genetics, Factor analysis

## Abstract

Multiple sclerosis (MS) is a heterogeneous neurological disorder with regards to clinical presentation and pathophysiology. Here, we investigated the heterogeneity of MS by performing an exploratory factor analysis on quantitative and qualitative neuropathology data collected for 226 MS donors in the Netherlands Brain Bank autopsy cohort. Three promising dimensions were identified and subsequently validated with clinical, neuropathological, and genetic data. Dimension 1 ranged from a predominance of remyelinated and inactive lesions to extensive pathological changes, higher proportions of active and mixed lesions, and foamy microglia morphology. This pattern was positively correlated with more severe disease, the presence of B and T cells, and neuroaxonal damage. Scoring high on dimension 2 was associated with active lesions, reactive sites, and the presence of nodules. These donors had less severe disease, a specific pattern of cortical lesions, and MS risk variants in the human leukocyte antigen region, the latter indicating a connection between disease onset and this neuropathological dimension. Donors scoring high on dimension 3 showed increased lesional pathology with relatively more mixed and inactive lesions and ramified microglia morphology. This pattern was associated with longer disease duration, subpial cortical lesions, less involvement of the adaptive immune system, and less axonal damage. Taken together, the three dimensions may represent (1) demyelination and immune cell activity associated with pathological and clinical progression, (2) microglia (re)activity and possibly lesion initiation, and (3) loss of lesion activity and scar formation. Our findings highlight that a thorough understanding of the interplay between multiple pathological characteristics is crucial to understand the heterogeneity of MS pathology, as well as its association with genetic predictors and disease outcomes. The scores of donors on the dimensions can serve as an important starting point for further disentanglement of MS heterogeneity and translation into observations and interventions in living cohorts with MS.

**Supplementary Information:**

The online version contains supplementary material available at 10.1007/s00401-024-02742-w.

## Introduction

Multiple sclerosis (MS) is one of the most common causes of severe neurological disability in early adulthood, resulting from a combination of environmental, lifestyle, and genetic factors [56, 62]. This chronic disease is characterised by lesions in the central nervous system, with varying extents of inflammation, demyelination, loss of axons, and gliosis [27]. MS is a highly heterogeneous disease, with contributions from concurrent pathophysiological processes that vary over time and between individuals [28]. As many different regions in the brain and spinal cord can be affected, MS is associated with a broad range of clinical signs and symptoms related to neurological dysfunction. Additionally, there is marked variation in disease course, severity, and response to disease-modifying treatment [25, 44]. Historically, MS has been divided into three clinical phenotypes: relapsing–remitting (RR), primary progressive (PP), and secondary progressive (SP) MS [30, 31]. This subdivision neither considers the possibility of relapses in progressive MS, nor progression independent of relapse activity in relapsing MS [3]. Therefore, the currently leading view is that this clinical division is rather artificial and that MS should be seen as one heterogeneous disease entity, best described in terms of relapse activity and progression [28, 30].

To address the heterogeneity of MS and the challenge this represents for allocating treatment and providing an accurate prognosis, multiple studies have defined MS subtypes based on underlying pathobiological mechanisms, using theory-driven as well as data-driven approaches. For example, analysis of magnetic resonance imaging (MRI) data led to the identification of two patient clusters, which were associated with different levels of clinical disability [51]. Another MRI study defined three subtypes, termed cortex-led, normal-appearing white matter-led, and lesion-led after the site of the earliest MRI abnormalities, which differed in the progression of disease and response to treatment [11]. Furthermore, a study investigating neuropsychological data found five distinct cognitive phenotypes, which were related to clinical, demographic, and MRI features and could be of added value in clinical care [10]. With regards to immunopathology, four patterns of demyelination in early, active MS lesions have been reported [32]. The patterns were heterogeneous between individuals, while lesions within the same brain generally followed the same patterns, even over time [32, 41]. This suggests that the four patterns could be used to define MS subgroups that correspond with distinct pathogenetic mechanisms involved in demyelination [32, 41]. However, this particular subdivision was not found in more advanced MS as studied at autopsy [2], in which the need for the development of new therapies is even more unmet than in primary stages, and which is the topic of this study.

Here, we aimed to discover dimensions of MS neuropathology, by performing an exploratory factor analysis of mixed data (FAMD) on post-mortem neuropathology data available from a large collection of MS brain donors in the Netherlands Brain Bank (NBB) autopsy cohort. The in-depth characterization of MS brain tissue facilitated a unique input dataset, containing information on the proportion of active, mixed active/inactive (mixed), inactive, and remyelinated white matter lesions, the morphology of microglia present in active and mixed lesions, the lesion load and reactive site load in the brainstem, the cortical lesion rate, and the presence of microglia nodules and perivascular cuffs. To investigate the validity of the FAMD dimensions, additional neuropathological, clinical, and genetic data available for this cohort were assessed. Key parameters that we included were related to disease progression and duration, signs and symptoms and their onset, grey matter lesions, the presence of B and T cells in the brainstem and subcortex, neuroaxonal damage, MS-associated genetic variants, and polygenic risk scores (PRSs).

## Methods

### NBB autopsy procedures and MS lesion characterization

NBB donors provided informed consent for brain autopsy and the use of tissue and data for research purposes. The procedures of the NBB are in compliance with Dutch and European law and have been approved by the Ethics Committee of the VU University Medical Center in Amsterdam, the Netherlands.

Both standardized locations in the brainstem and spinal cord as well as macroscopically detectable MS lesions were dissected, as previously described by Luchetti et al. [33]. Since 2001, lesions were additionally dissected under guidance of post-mortem MRI, which increased the yield of active demyelinating lesions and reactive sites [9]. Lesions were classified based on double immunostaining for proteolipid protein and human leukocyte antigen (HLA-DR-DQ) [33]. In total, five qualitatively different white matter anomalies and/or lesion types were distinguished: (1) reactive sites, clusters of HLA^+^ microglia/macrophages without signs of demyelination; (2) active lesions, with partial demyelination and accumulation of HLA^+^ cells throughout the lesions; (3) mixed lesions, with a demyelinated, hypocellular, and gliotic centre and a rim of HLA^+^ cells; (4) inactive lesions, a fully demyelinated, hypocellular, and gliotic region with no accumulation of HLA^+^ cells; and (5) remyelinated lesions, with partial myelination and few HLA^+^ cells. The HLA^+^ microglia/macrophages in active and mixed lesions were additionally scored for morphology: (1) thin and ramified; (2) ameboid (rounded) with few ramifications; and (3) foamy and lipid-laden. In the remainder, these HLA^+^ microglia/macrophages will be referred to solely as ‘microglia’. Grey matter lesions were dissected as well, and classified according to their location into: (1) leukocortical, located in both white and grey matter; (2) intracortical; and (3) subpial. Lesions were only considered subpial if the tissue block contained the first layer of the cortex, with the consequence that the number of intracortical lesions is overestimated to some extent. Some (large) cortical lesions may have been sampled and counted multiple times.

Lesion load was previously defined as the total number of active, mixed, inactive, and remyelinated lesions present in standardly dissected brainstem tissue blocks [13, 14, 23, 33, 58, 59]; similarly, reactive site load was calculated as the total number of reactive sites in these brainstem blocks [33, 59]. These values are therefore not affected by sampling bias and considered to be an adequate reflection of the total number of white matter lesions and reactive sites present in a donor, superior to a lesion rate calculated with data from all dissected tissue blocks. Furthermore, the proportions of active, mixed, inactive, and remyelinated lesions relative to the total number of white matter lesions in all dissected tissue blocks were calculated. In addition, the tissue blocks were assessed to determine the presence of cuffs (defined as > 1 perivascular ring of leukocytes) and microglia nodules. This was converted into binary scores per donor, indicating the overall presence or absence of cuffing and nodules, respectively. As before, the microglial/macrophage activation score was calculated by assigning scores to the predominant morphology of microglia in active and mixed lesions (ramified = 0, ameboid = 0.5, and foamy = 1) and averaging these scores per donor [33].

With regards to grey matter, the cortical lesion rate was previously defined as the number of cortical lesions per tissue block containing cortex [23]. Note that in contrast to the white matter lesion load, this value can be affected by sampling bias. If one or more cortical lesions were present, the relative proportions of the different grey matter lesion types were determined.

### Identifying dimensions of MS neuropathology

A centered log ratio (CLR) transformation was applied to the proportional lesion data, to reduce the possibility of correlations arising solely from the constant sum constraint (i.e. for each donor, the values of the proportions sum to 1) [18]. The CLR transformation takes the logarithm of each lesion proportion, relative to the geometric mean of all lesion proportions for a donor; because this requires non-zero data, multiplicative zero replacement was performed beforehand [38]. Both were implemented using the package Compositional (v6.3) with R version 4.1.3 [49].

Initially, analysis was restricted to donors for whom complete proportional white matter lesion data was available (n = 228). To maximize the number of observations in the dataset, missing data in other variables was accepted. For 14 donors, lesion load and reactive site load were missing, most probably because no standard locations were dissected; data on the cortical lesion rate was missing in 25 cases, likely because no blocks with cortical tissue had been dissected. All missing values were imputed with the function imputeFAMD from the missMDA package (v1.18) [24], based on the predicted optimal number of dimensions returned by estim_ncpFAMD (Kfold cross-validation with standard settings, with the maximum set at 12). The imputed dataset consisted of 228 observations for 13 neuropathology-related variables: transformed white matter lesion proportions, lesion load, reactive site load, cortical lesion rate, and the presence or absence of cuffing and nodules.

Next, a first FAMD (factor analysis of mixed data) was performed using the FactoMineR package (v2.8) [29], which scales continuous and categorical variables in order to balance their impact on the analysis. Of the 12 dimensions comprising the FAMD output, a subset was selected for further investigation based on an objective criterion (eigenvalue > 1.5). Highly contributing outliers were defined as donors that a) contributed substantially to one or more dimensions, i.e. had a contribution larger than the upper cutoff value for the upper whisker of an adjusted boxplot, and b) that were a univariate outlier with regards to the continuous input variables, i.e. had (a) value(s) lower than the lower cutoff value or higher than the upper cutoff value for the whiskers of an adjusted boxplot. Using the adjusted boxplot method ensured that the skewness of the distributions was taken into account when detecting outliers [21]. Donors meeting both conditions a and b were removed from the input dataset, which was followed by further rounds of imputation, FAMD, and outlier detection and removal, until no more highly contributing outliers were identified; one round was needed and led to removal of two donors. Altogether, this procedure reduces the impact of individual donors (and variables) on the analysis and thereby results in more robust dimensions that capture shared neuropathological patterns.

### Validation and exploration with additional neuropathology, clinical, and genetic data

To interpret the meaning and validity of the dimensions, relevant external data which was not used as input is of key importance. Here, we assessed whether the dimensions displayed associations with neuropathological, clinical, and genetic data for validation purposes, and explored associations with comorbidities and drug use.

#### Description of general clinical data

General clinical information was obtained by retrospective chart analysis for the majority of the donors [33]. Clinical course was extracted from the patient files, categorised by the treating neurologist in clinical practice into relapsing (including both progressive relapsing and relapsing–remitting MS), progressive with a relapsing onset (SP) or progressive without dominant relapsing onset (PP). These phenotypes were reviewed by an independent neurologist. Other characteristics determined in this manner were age at onset, time from onset to (estimated) Expanded Disability Status Scale (EDSS)-6 (i.e. needing a walking aid), and duration of disease.

#### Analysis of the frequency and onset of signs and symptoms

For a complete description of the processing of clinical text data to develop disease trajectories for donors in the NBB autopsy cohort, see Mekkes et al. [40]. Briefly, state-of-the-art natural language processing techniques were used in order to identify the presence of 84 signs and symptoms (attributes) in individual sentences of medical record summaries. This led to a high-quality dataset of clinical disease trajectories, allowing researchers to study the clinical manifestation of signs and symptoms across disorders. For this study, we investigated the frequency and age at onset of the attributes, grouped together into the five overarching domains defined by Mekkes et al. [40]: general, motor, sensory/autonomic, cognitive, and psychiatric. The attribute definitions, ontology structure, and clinical disease trajectories of NBB donors are accessible via https://nnd.app.rug.nl. Suppl. Figure 1 (Online Resource 1) shows how often individual attributes were observed in our final cohort (after outlier removal), how many donors experienced the attribute at least once, how often an attribute was observed within the lifetime of these donors, and the ages at which the attribute was observed. To assess the symptom load of donors per domain, the total number of observations (in a donor’s lifetime) of all attributes within the domain was divided by the disease duration of the donor. In addition, we determined the median age at onset per domain, by identifying all ages at which the attributes within a domain were first observed and taking the median value.

#### Analysis of comorbidities

Lifetime diagnosis information was available from the study by Mekkes et al. [40]. In short, clinical diagnoses of NBB donors were parsed and manually matched to classes of the Netherlands Neurogenomics Database Human Disease Ontology (NND-HDO, accessible via https://nnd.app.rug.nl or BioPortal (https://bioportal.bioontology.org/ontologies/NND_CD)). In the following, ‘category’ refers to a higher-level class together with its subclasses, while ‘class’ is used to indicate only the class itself. For relevant diagnosis categories, we determined how often that category was observed in our final cohort, how many donors had at least one observation of the category, and the years and ages at which the category was observed; the most commonly observed classes in the MS cohort were analysed similarly (suppl. Figure 2, Online Resource 1). If a class was observed multiple times for a donor, only the class observation at the earliest time point was included. To filter out incomplete files and restrict our analysis to comorbidities, donors with no observations of classes other than MS or its subclasses were not considered in our analysis. The relation between comorbidities and the dimensions, year of death, and age at death was investigated for both relevant categories and common classes by comparing donors with and without at least one observation of the category or class, respectively.

#### Analysis of drug use

Medication data was extracted from the medication summaries in the NBB donor files using a system of text-parsers, and then preprocessed by extracting timepoints, removing non-medication text (including dosages), and manually resolving spelling mistakes and ambiguity. Elements of combination drugs (separated with a slash) were considered individually (e.g. miconazole/hydrocortisone), and common drug names consisting of multiple words (e.g. interferon beta-1a) were manually transformed into one word (connected with dashes). The resulting drug texts were matched to standard Anatomical Therapeutic Chemical (ATC) codes using the MOLGENIS SORTA parser (version 10.1.0) [45, 60], modified for drug to ATC matching by Kellmann et al. [26], with the confidence threshold for the similarity score set to 78.5%. For each timepoint, higher ontology level ATC codes were removed if a more specific ATC code (within that same class) was matched as well, to prevent double matching/counting of drug texts. The timepoints were then categorised into ‘last hours’, ‘death year’, ‘earlier’, and ‘unknown’, and ATC codes were binarized at the level of these categories (i.e. drugs were considered to be observed/not observed per time category). Donor files with ≥ 200 characters and ≥ 25 words in the medication summary that contained observations in both the year of death (i.e. either ‘last hours’ or ‘death year’) and another year (i.e. ‘earlier’ or ‘unknown’) were considered high quality and were included in our analysis. For all ATC level 1 (L1) classes and a subset of level 2 (L2) classes, we determined how often and at which time category the drug class was observed, and how many donors had at least one observation (suppl. Figure 3a, Online Resource 1). To explore the relation between these drug classes and the dimensions, year of death, and age at death, donors with and without at least one observation of the class were compared, excluding observations in the ‘last hours’ category. In addition, to address potential confounding of the dimensions by the use of MS-relevant drug therapies, we composed a list with (1) the most commonly observed drugs (over all time categories), (2) the drugs most commonly used by the donors, (3) all drugs within the L1 class ‘NERVOUS SYSTEM DRUGS’, and (4) all drugs within the L1 class ‘ANTINEOPLASTIC AND IMMUNOMODULATING AGENTS’. For each drug on this list (n = 228 unique drugs, of a total of 585 unique drugs observed in the high-quality files), we then determined if it was a disease-modifying or immunosuppressive drug prescribed for MS. Suppl. Figure 3b (Online Resource 1) shows an overview of the number of observations and donors using these drugs. Potential confounding was assessed by comparing the scores on the dimensions of donors with and without at least one observation of the disease-modifying or MS-relevant immunosuppressive drug categories; the association with year of death and age at death was analysed similarly.

#### Description of neuropathological data

Whereas the cortical lesion rate was part of the input dataset, the proportions of grey matter lesion types were used for validation. Similar to the white matter lesion proportions, a CLR transformation was applied before analysing the relation with the dimensions. Furthermore, information on the presence of B cells—either diffusely or as part of (an) infiltrate(s)—in the medulla oblongata was available for 132 donors from a previous study by Fransen et al., which noted that B cell presence at various locations was correlated and could thus be considered a general donor characteristic [13]. We also analysed their data on the presence of B cells in different white matter lesion types in the subcortex when data was available for > 20 donors in our final cohort [13]. The number of CD3^+^ cells per mm^2^ in normal-appearing white matter (NAWM) of the pyramidal tract at the level of the medulla oblongata and in subcortical (perilesional) NAWM was previously determined by Fransen et al. [14], and was available for 95 and 53 donors included in this study, respectively. CD3^+^ cell counts in subcortical white matter lesions derived from the same study were analysed when information was available for > 20 donors [14]. Van den Bosch et al. examined the extent of neuroaxonal damage by measuring neurofilament light chain (NfL) levels in cerebrospinal fluid (CSF), axonal density in the NAWM of the pyramid tract by determining the percentage of Bielschowsky positive area, and acute axonal stress by detecting the presence of amyloid precursor protein (APP) positive axonal fragments or bulbs in subcortical (perilesional) NAWM [58]; this data was available for 99, 55 and 52 of our donors, respectively. Donors with a stroke in the year before death, a clinically silent stroke, or brain atrophy were excluded from the analysis of CSF NfL levels (n = 35 excluded, n = 64 remained), because having a recent or silent stroke or atrophy could confound this data [58].

#### Genotyping of the NBB autopsy cohort, quality control (QC) and imputation

Samples derived from donors in the NBB autopsy cohort were genotyped with the Infinium Global Screening Array (Illumina, v3) by the Human Genomics Facility at Erasmus Medical Centre. The Human Genomics Facility also performed initial processing of the data, using the PLINK toolset [48]. Pre-imputation QC consisted of iterative removal of variants and samples with missing data so that the final call rate exceeded 97.5% (99% after zCall), removal of variants deviating from Hardy–Weinberg equilibrium (HWE) with excess heterozygosity (P < 1 × 10^–5^), and exclusion of samples with excess heterozygosity (inbreeding coefficient F_sample_ < mean F − 4 × standard deviation (SD)). Genetic duplicates and potential sample swaps were identified and removed. To improve detection of rare variants, zCall was calibrated and applied [17], followed by QC using the thresholds specified before and removal of duplicate variants. Principal component analysis on ancestry informative markers of samples of the NBB cohort and the 1000 Genomes Project (phase 3, v5 [55]) was used to identify NBB donors with non-European ancestry (deviating > 4 × SD from the mean of the European reference dataset on the first four principal components), which were excluded from further analysis. Correction for first- and second-degree familial relationships within the cohort was performed with KING software [37]. Imputation was done in a two-step procedure using SHAPEIT for phasing and Minimac4 for imputation to the HRC (Haplotype Reference Consortium) r1.1 reference panel [7, 39].

#### Processing of imputed genetic data and calculation of PRSs (polygenic risk scores)

Imputed genetic data was processed and analysed using PLINK 2.0 software (v2.00a4LM (3 Mar 2023)) [5]. Variants and samples with a missing call rate > 0.05 were removed. Variants with a nonmajor allele frequency < 0.05, variants with a low or medium quality of imputation (R2 < 0.8) and variants deviating from HWE (*p* < 1 × 10^–6^) were filtered out as well. Ultimately, genetic information was available for 194 donors of the final cohort studied here. Since our cohort size precludes an extensive genetic analysis, we decided to focus on two variants: rs3135388, the tagging single nucleotide polymorphism (SNP) of the HLA-DRB1*15:01 allele, which confers a greatly increased susceptibility to develop MS [8, 19], and rs10191329, the SNP that was significantly associated with the age-related MS severity score in a recent genome-wide association study (GWAS) [23]. A standard association analysis with the ranked score of genotyped donors on the dimensions was performed with PLINK 2.0 using the command –glm, which fits a linear regression model for each variant. In addition, MS PRSs were constructed using the LDAK-BayesR-SS tool [65]. We implemented QuickPRS to construct a prediction model based on the most recent MS GWAS by Patsopoulos et al. [47]. To assess the relation with autosomal MS-associated variants outside the HLA region, a non-HLA MS PRS was calculated by excluding the extended HLA region on chromosome 6 as defined previously by Patsopoulos et al. [47] (base pairs 24,000,000 to 35,000,000; genome assembly GRCh37/hg19).

### Statistical analysis

All statistical analyses were performed in Python (v3.8.8). To assess the association between the dimensions and continuous validation variables, non-parametric Spearman correlation was performed with Scipy (v1.10.1). The Spearman correlation coefficient is denoted as ‘rho’ in figures and text. The association with categorical variables was tested by performing non-parametric Kruskal–Wallis and/or (post-hoc) Mann–Whitney U tests with Scipy. To correct for multiple testing, the Benjamini–Hochberg—False Discovery Rate (FDR) was implemented using the statsmodels package (v0.14.0); the significance threshold was set to *p* ≤ 0.1. FDR correction was performed for all tests between the different dimensions and one variable or a set of closely related variables (i.e. age at MS onset, age at death, years till EDSS-6, and disease duration; symptom load for the different domains; median age at onset per domain; clinical diagnosis categories and classes; drug use for L1 and L2 ATC classes; use of disease-modifying and MS-relevant immunosuppressive drugs; cortical lesion proportions; presence/absence of CD20^+^ cells, CD138^+^ cells, and lesions in the brainstem; presence/absence of CD20^+^ cells in subcortical lesions; presence/absence of CD138^+^ cells in subcortical lesions; number of CD3^+^ cells in subcortical NAWM and lesions; the two genetic variants). To clearly show the direction and strength of the association between dimensions and continuous variables, a regression line was plotted on top of a scatter plot of the ranked data; the slope and intercept were calculated with Scipy, by performing linear least-squares regression on the ranked data. Raw input data and (anonymised) validation data is provided for 228 donors (including the two highly contributing outliers) in Online Resource 2.

## Results

### Dimensionality reduction to disentangle the heterogeneity of MS neuropathology

**Fig. 1:**
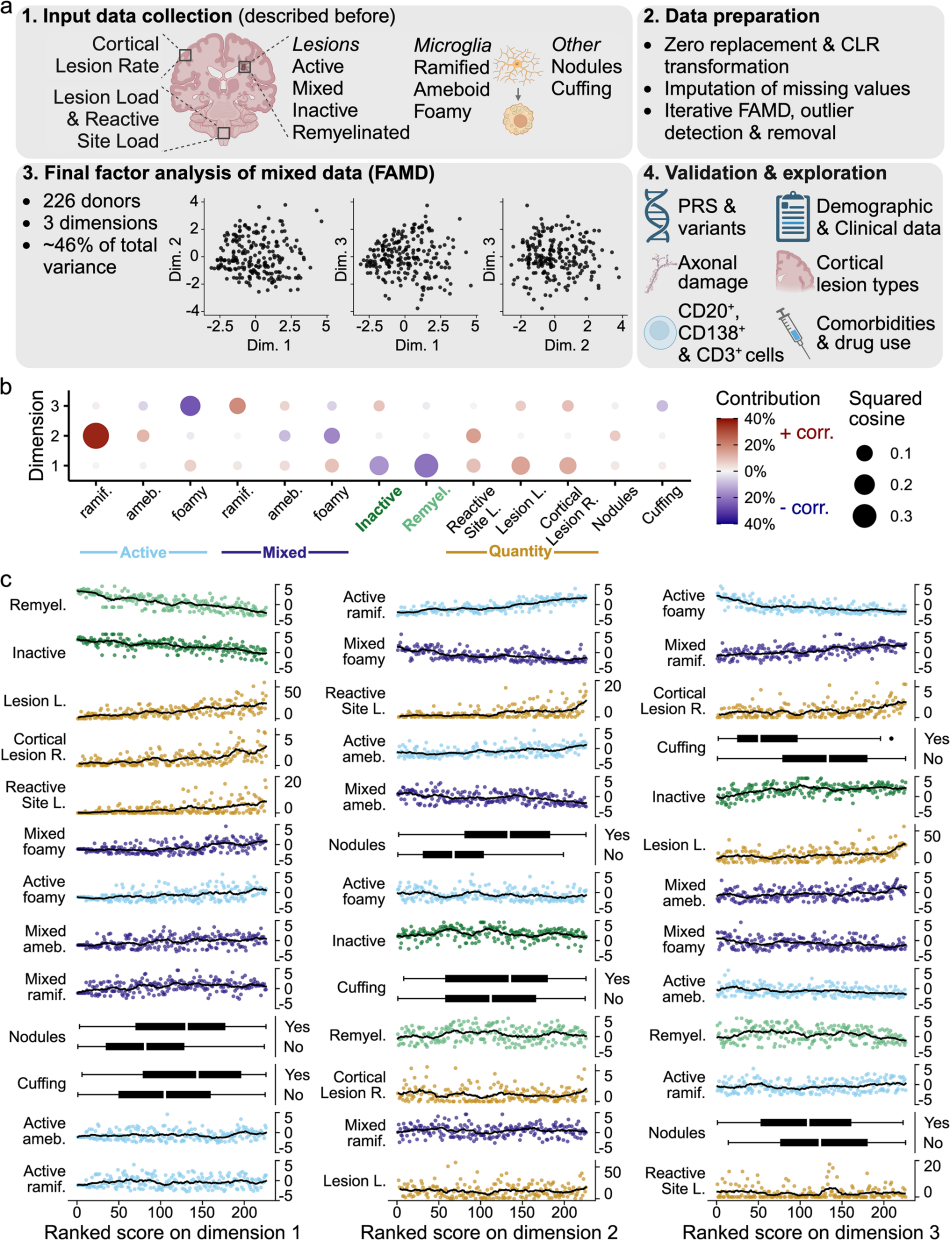
Overview of the study and the three independent dimensions. **a** Graphical outline of this study created with BioRender.com, consisting of 4 stages: (1) collecting previously generated input data, (2) data preparation, (3) exploratory FAMD, with scatter plots showing how donors score on the resulting dimensions, and (4) validation and exploration. **b** Dot plot displaying the relation between FAMD input variables and the first three dimensions. Dot size indicates the squared cosine, reflecting the proportion of variance in a variable explained by a dimension. Higher values correspond to a better quality of representation of the variable by the dimension. Dot color indicates whether the correlation between the dimension and the variable is positive (red) or negative (blue); color intensity reflects the relative contribution of the variable to the component. **c** Scatter plots with fitted line and box plots per dimension, depicting the values for the input variables on the *Y*-axis, for donors ranked according to their score on each dimension on the *X*-axis. In case of ties, donors were assigned ranks in the order of appearance in the dataset, so that each donor receives a unique rank. The line represents the centered moving average, over a window of 20 observations, with a maximum of 10 missing values. Proportional data is CLR-transformed; unimputed values are shown. Plots are ordered vertically based on the variable’s contribution to the dimension. CLR = centered log ratio; FAMD = factor analysis of mixed data; dim. = dimension; PRS = polygenic risk score; ramif. = ramified; ameb. = ameboid; remyel. = remyelinated (lesion); L. = load; R. = rate; corr. = correlation

To identify patterns in MS neuropathology, we established a computational workflow that consists of four stages (Fig. 1a). In stage 1, we collected previously generated quantitative and qualitative neuropathological data, predominantly related to the white matter [33]. Stage 2 consisted of processing this data, including transformation, imputation and an initial FAMD (factor analysis of mixed data). We selected the first three dimensions for further analysis and subsequently removed two donors with outlier values who contributed highly to a dimension. In stage 3, FAMD was performed on the definite dataset of 13 variables across 226 donors. Of the final FAMD dimensions, the first three together accounted for almost half of the total variance in the dataset, indicating that closer examination could provide insight into general neuropathological patterns observed in our MS cohort. In stage 4, we used orthogonal data types (clinical [33, 40], neuropathological [33, 58], immunological [13, 14], and genetics) to evaluate and interpret the dimensions.

First, we explored the relation between input variables and the three dimensions identified in stage 3. Each dimension reflected a distinct neuropathological pattern, in which the variables were represented by and contributing to each dimension to varying extents (Fig. 1b). In addition, we ranked all donors according to their score on each dimension (in ascending order, from low to high) to illustrate how the dimensions were related to the values of the input variables (Fig. 1c). Critically, donors’ scores on one dimension were independent of their scores on the other dimensions—the dimensions are uncorrelated (as shown by the scatter plots in Fig. 1a). A low score on dimension 1 was associated with a predominance of inactive and remyelinated lesions with few HLA^+^ microglia/macrophages, a high score with a high lesion load, active and mixed lesions populated by foamy microglia, and the presence of nodules and cuffs (perivascular accumulations of leukocytes). Dimension 2 was positively correlated with the proportion of active lesions, the number of reactive sites, and the presence of nodules. Dimension 3 was positively associated with a higher cortical lesion rate and lesion load, a larger proportion of mixed lesions with ramified microglia and inactive lesions, relatively fewer active lesions with foamy microglia, and an absence of cuffs.

Next, we aimed to validate and explore the dimensions. For an overview of the relation between the dimensions and all variables analysed in this study, see Table 1.

**Table 1:** Overview of the relation between the dimensions and variables analysed in this study

	Dimension 1	Dimension 2	Dimension 3
Interpretation	Immune cell activity & demyelination related to progression	Microglia (re)activity & possibly lesion initiation	Loss of lesion activity & scar formation
Eigenvalue (% var.)	2.60 (19.98%)	1.70 (13.06%)	1.67 (12.83%)
*Input variables*			
Lesion quantity	More lesions in brainstem & cortex, more reactive sites	More reactive sites	More lesions in brainstem & cortex
Lesion type	More active & mixed, less inactive & remyelinated	More active, less mixed	More mixed & inactive, less active & remyelinated
Microglia	More foamy	More ramified	More ramified
Cell clusters	More often cuffs & nodules	More often nodules	Less often cuffs
*Demographic*			
Sex	ns	ns	ns
Age at death	Younger at death (****)	Older at death (***)	ns
*Clinical*			
MS type	ns	ns	ns
Severity	Shorter time from onset to EDSS-6 (****) & death (****)	Longer time from onset to EDSS-6 (**) & death (****)	Longer time from onset to death (*)
Symptom load per domain	More general (**), motor (*), sens. (*) & cogn. (+) observations	Less general (+), motor (+), sens. (+) & psych. (*) observations	Less sens. (+) & psych. (+) observations
Age at MS onset	ns	ns	ns
Median age at domain onset	Younger at onset of general (****), motor (****), sens. (****), cogn. (****) & psych. (****) domains	Older at onset of general (**), motor (+), sens. (*), cogn. (**) & psych. (+) domains	Older at onset of cogn. domain (+)
Comorbidities	Less cardiovascular system dis. (**), hypertension (*) & autoimmune dis. (*)	More diabetes mellitus type 2 (*)	ns
Drug use	More disease-modifying drug use (*)	Less use of MS-relevant immunosuppressants (*)	ns
*Genetics*			
MS PRS	ns	Higher MS PRS (**)	ns
rs3135388	ns	Higher risk allele dosage (**)	ns
rs10191329	ns	ns	ns
*Cortical lesions*			
Presence	More often present (****)	ns	More often present (*)
Lesion type	Less intracortical (**)	Less leukocortical (***), more intracortical (*) & subpial (*)	More subpial (*)
*Neuroaxonal damage*			
CSF NfL	Higher levels (+)	ns	Lower levels (*)
Bielschowsky positive area	ns	ns	Higher density (+)
APP^+^	ns	ns	ns
*Immune cells*			
CD20^+^	More in pch (*) & pvs (+), more in active (*) & mixed (*) lesions	ns	Less in active (+) and mixed (+) lesions
CD138^+^	More in pch (**) & pvs (*)	ns	Less in pch (*)
Lesion in MOB block	More often with lesion (****)	ns	ns
CD3^+^	More in subcortical NAWM (*) & active lesions (+)	ns	ns
*Other*			
Autopsy year	ns	More recent (****)	More recent (**)
CoD	ns	*	ns
pH, pmd & weight	ns	ns	ns

Note that the correlations are described for one direction, from low to high rank/score on the respective dimension. var. = variance; EDSS = Expanded Disability Status Scale; sens. = sensory/autonomic; psych. = psychiatric; cogn. = cognitive; dis. = disease; CSF = cerebrospinal fluid; NfL = neurofilament light chain; APP = amyloid precursor protein; pch = parenchyma; pvs = perivascular space; MOB = medulla oblongata; CoD = cause of death; pmd = post-mortem delay; ns *p* > 0.1; + *p* ≤ 0.1; * *p* ≤ 0.05; ** *p* ≤ 0.01; *** *p* ≤ 0.001; **** *p* ≤ 0.0001

### Dimensions are associated with year of autopsy and cause of death

The dimensions were not associated with post-mortem delay, pH or brain weight (suppl. Figure 4, Online Resource 1), implying that they were not driven by these technical covariates. Donors who scored high on dimension 2 or 3 were likely to have died in more recent years (dim. 1: *p* = 0.17, rho = 0.09; 2: *p* = 3.5 × 10^–5^, rho = 0.29; 3: *p* = 0.006, rho = 0.19; suppl. Figure 5, Online Resource 1). There was no association with cause of death, except for dimension 2 (dim. 1: *p* = 0.12, 2: *p* = 0.05, 3: *p* = 0.51; suppl. Figure 6, Online Resource 1); donors who died by legal euthanasia scored higher on this dimension than donors who died of unspecified natural causes (*p* = 0.02). This finding could reflect an association between year and cause of death, possibly related to Dutch legislation on euthanasia taking effect in 2002. Accordingly, the median year of autopsy was 2013 for donors who died by euthanasia and 2005.5 for those who died from unspecified natural causes.

### Dimensions are associated with the clinical manifestations of MS

**Fig. 2:**
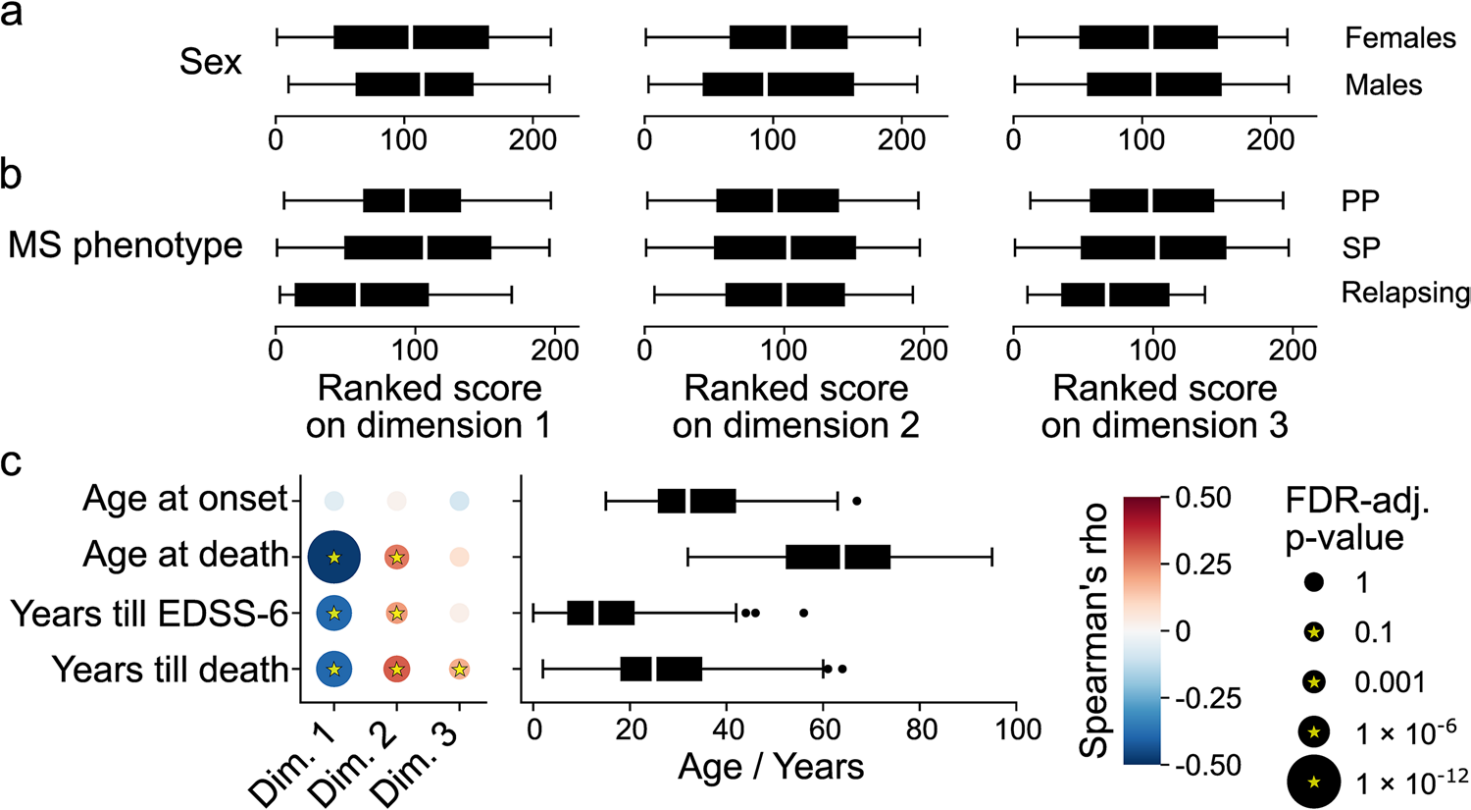
Association between demographic and clinical variables and dimensions. Note that *X*-axis scale differs among the different plots. **a** Box plots showing the ranked scores of donors, per dimension, per sex. Sex was known for 214 donors (77 males, 137 females). There are no significant differences between sexes (Mann–Whitney U; dim. 1: *p* = 0.80; 2: *p* = 0.80; 3: *p* = 0.93). **b** Box plots showing the ranked scores of donors, per dimension, per MS clinical phenotype. MS phenotype was determined for 197 donors (12 relapsing, 119 SP, 66 PP). There are no significant differences (Kruskal–Wallis; dim. 1: *p* = 0.30; 2: *p* = 0.88; 3: *p* = 0.30). In plots in **a** and** b**, ties were assigned averaged ranks. **c** Dot plot (left) showing the correlation between the scores on dimensions 1–3 and age at MS onset for 204 donors, age at death for 214 donors, years till EDSS-6 for 191 donors, and years till death (i.e. duration of disease) for 201 donors. Dot colour indicates the strength of the correlation, dot size the *p*-value, and a yellow star a significant association. Box plots (right) showing the distribution of the demographic and clinical variables. Significance in **c** was assessed with Spearman correlation and FDR-adjusted for multiple testing. dim. = dimension; PP = primary progressive; SP = secondary progressive; EDSS = Expanded Disability Status Scale; FDR = False Discovery Rate

To investigate the potential clinical correlates of the neuropathological dimensions, we subsequently focused our analysis on data related to demographics and disease experience. There was no significant correlation with sex (dim. 1: *p* = 0.80; 2: *p* = 0.80; 3: *p* = 0.93) or MS clinical phenotype (dim. 1: *p* = 0.30; 2: *p* = 0.88; 3: *p* = 0.30) (Fig. 2a, b), the latter supporting the notion that the historically identified clinical MS phenotypes do not qualitatively differ with regards to the (white matter) pathological features included in our analysis.

Dimension 1 was negatively associated with the years from onset till EDSS-6 (*p* = 7.7 × 10^–8^, rho = −0.39), from onset till death (i.e. duration of disease; *p* = 5.8 × 10^–8^, rho = −0.39), and age at death (*p* = 1.6 × 10^–12^, rho = −0.48); there was no relation with age at MS onset (*p* = 0.51, rho = −0.06) (Fig. 2c). In addition, the total number of signs and symptoms adjusted for disease duration (the symptom load) was significantly positively correlated with dimension 1 for the general, motor, sensory/autonomic, and cognitive domains (suppl. Figure 7, Online Resource 1). Corresponding with their earlier death, the median age at symptom onset was generally earlier for donors scoring high on dimension 1 (suppl. Figure 8, Online Resource 1). On the whole, a higher score on dimension 1 associates with a more severe disease course of MS.

In contrast, dimension 2 was associated with milder MS, positively correlating with the years till EDSS-6 (*p* = 0.006, rho = 0.21), till death (*p* = 4.3 × 10^–5^, rho = 0.30), and age at death (*p* = 2.4 × 10^–4^, rho = 0.26) (Fig. 2c). Age at onset was not associated with the dimension (*p* = 0.78, rho = 0.02; Fig. 2c). Moreover, for all domains the symptom load was negatively correlated with dimension 2, although not always significantly so (suppl. Figure 7, Online Resource 1). The median age at symptom onset was positively correlated with dimension 2, in line with expectations (suppl. Figure 8, Online Resource 1).

Dimension 3 was not significantly correlated with years till EDSS-6 (*p* = 0.74, rho = 0.03), age at MS onset (*p* = 0.31, rho = −0.09), and age at death (*p* = 0.37, rho = 0.07) (Fig. 2c). However, because donors who scored high on dimension 3 tended to develop MS at a slightly younger age and died at a relatively older age, there was a significant positive correlation with disease duration (*p* = 0.02, rho = 0.18; Fig. 2c). Interestingly, with regards to the symptom load, the overarching domain might matter: there was a significant negative correlation for the psychiatric and sensory/autonomic domains only (suppl. Figure 7, Online Resource 1). Furthermore, there was a positive association with median age at symptom onset for the cognitive domain (suppl. Figure 8, Online Resource 1).

The identified dimensions correlate with clinical profiles of NBB MS donors, and therefore likely reflect clinically relevant pathological processes. In the following sections, each dimension will be further examined with regards to comorbidities, drug use, cortical neuropathology, potential immune cell involvement, and genetics.

### Dimensions correlate with certain comorbidities and use of MS-relevant drugs

To assess associations between our dimensions and comorbidity, we compared the MS donors with and without other diagnoses (i.e. with or without one or more observations of relevant categories and classes); similarly, we compared the donors who did and did not use a particular class of drugs. MS donors with an autoimmune disease scored significantly lower on dimension 1. Donors with a cardiovascular system disease or its subclass hypertension had a higher age at death and scored lower on dimension 1 (suppl. Figure 9, Online Resource 1). Consistently, donors who used cardiovascular system drugs died at an older age and scored lower on dimension 1 (although the latter was not significant after multiple-testing correction; suppl. Figure 10, Online Resource 1), corroborating the quality of our datasets. Type 2 diabetes mellitus was more common in older donors, and donors with this comorbidity scored significantly higher on dimension 2 (suppl. Figure 9, Online Resource 1). Regarding drug therapies relevant for MS, donors using disease-modifying drugs scored higher on dimension 1, died at a younger age, and died more recently. Donors who used MS-relevant immunosuppressive drugs generally scored lower on dimension 2, died at a younger age, and in less recent years (suppl. Figure 11, Online Resource 1).

### Dimension 1: demyelination with an active immune system

**Fig. 3:**
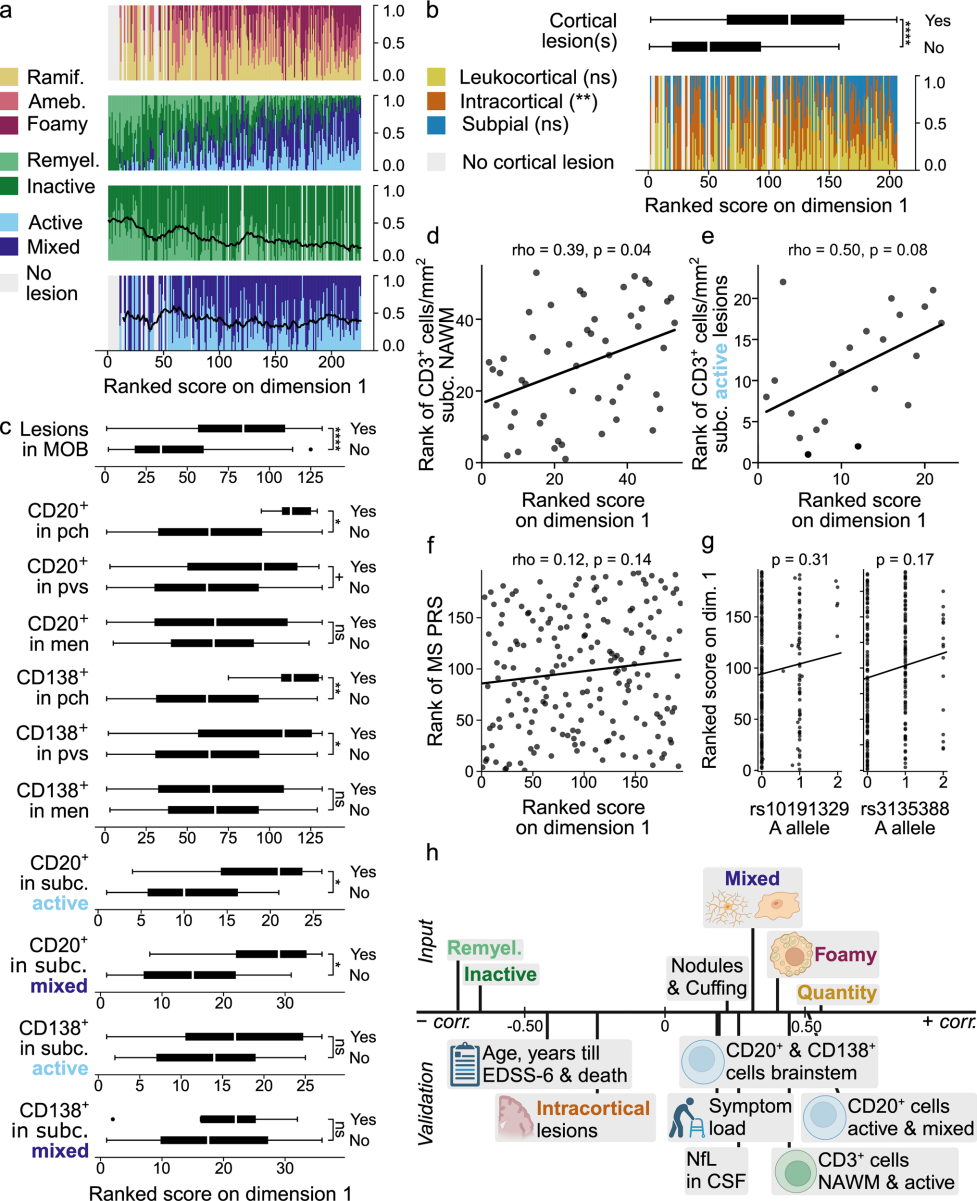
Dimension 1. **a** Vertical bar graphs showing on the *Y*-axis the ramified, ameboid and foamy microglia proportions relative to the total number of active and mixed lesions, white matter lesion proportions relative to the total number of white matter lesions, remyelinated and inactive lesion proportions relative to the total number of end-stage (remyelinated plus inactive) lesions, and active and mixed lesion proportions relative to the total number active plus mixed lesions, from top to bottom respectively, with donors ranked according to their score on the *X*-axis. The lines represent the centered moving average of the ratio remyelinated to end-stage, and the ratio active to active plus mixed, over a window of 20 observations, with a maximum of 10 missing values. **b** Box plot (top) showing the ranked scores for 175 donors with and 31 donors without (a) cortical lesion(s). Vertical bar graph (bottom) showing the proportions of cortical lesion types on the *Y*-axis for donors ranked according to their score on dimension 1. In **a** and **b**, ties were assigned ranks in the order of appearance in the dataset. **c** Box plots showing, from top to bottom, the ranked scores of 83 donors with and 49 without lesions in the MOB tissue block used to determine B cell presence; 6 donors with and 126 without CD20^+^ cells in pch; 21 donors with and 111 without CD20^+^ cells in pvs; 69 donors with and 63 without CD20^+^ cells in men; 9 donors with and 123 without CD138^+^ cells in pch; 14 donors with and 118 without CD138^+^ cells in pvs; 62 donors with and 70 without CD138^+^ cells in men; 10 donors with and 16 without CD20^+^ cells in subc. active lesions; 11 donors with and 25 without CD20^+^ cells in subc. mixed lesions; 6 donors with and 21 without CD138^+^ cells in subc. active lesions; and 4 donors with and 32 without CD138^+^ cells in subc. mixed lesions. **d**, **e** Scatter plots with regression lines, depicting on the *Y*-axis the rank of the CD3^+^ cell count in subc. NAWM for 53 donors (**d**) and in subc. active lesions for 22 donors (**e**), with donors ranked according to their score on dimension 1 on the *X*-axis. **f** Scatter plot with regression line, depicting the rank of the MS PRS (polygenic risk score) on the *Y*-axis for 194 donors, ranked according to their score on dimension 1 on the *X*-axis. **g** Two scatter plots with regression lines showing the ranked score of donors on dimension 1 on the *Y*-axis, and allele dosage on the *X*-axis. In **c–g**, ties were assigned averaged ranks. **h** Overview of the correlation between relevant input and validation variables and dimension 1, created with BioRender.com. Position on the axis is a close approximation of the Spearman correlation between the dimension and the variable(s); variables were grouped when appropriate and positioned based on the average of the correlation coefficients. Significance in **b–f** was assessed with Mann–Whitney U for binary variables and Spearman correlation for continuous variables and FDR-adjusted for multiple testing; for **g** see text. ns *p* > 0.1; + *p* ≤ 0.1; * *p* ≤ 0.05; ** *p* ≤ 0.01; *** *p* ≤ 0.001; **** *p* ≤ 0.0001; ramif. = ramified; ameb. = ameboid; remyel. = remyelinated (lesion); MOB = medulla oblongata; pch = parenchyma; pvs = perivascular space; men = meninges; subc. = subcortical; NAWM = normal-appearing white matter; dim. = dimension; EDSS = Expanded Disability Status Scale; CSF = cerebrospinal fluid; NfL = neurofilament light chain; FDR = False Discovery Rate

Microglia morphology was strongly associated with dimension 1, in the form of a positive correlation with the microglial/macrophage activation score (suppl. Figure 12, Online Resource 1); the associated lesion type (active or mixed) seemed to be of less importance. End-stage (remyelinated and inactive) lesions were relatively infrequent in donors with a high score on dimension 1 (Fig. 3a).

Donors with (a) cortical lesion(s) scored higher on dimension 1 (*p* = 3.8 × 10^–6^). Moreover, donors who scored high on dimension 1 had relatively fewer intracortical lesions (*p* = 0.006, rho = −0.24). Although dimension 1 was correspondingly positively correlated with the proportion of leukocortical and subpial lesions, this did not reach statistical significance (leukocortical: *p* = 0.26, rho = 0.09; subpial: *p* = 0.22, rho = 0.10) (Fig. 3b & suppl. Figure 13, Online Resource 1).

Donors with CD20^+^ and/or CD138^+^ cells in the parenchyma and/or perivascular space of the brainstem scored higher on dimension 1 (CD20^+^ parenchyma (pch): *p* = 0.01; CD20^+^ perivascular space (pvs): *p* = 0.06; CD138^+^ pch: *p* = 0.001; CD138^+^ pvs: *p* = 0.04). Furthermore, presence of CD20^+^ cells in active and mixed subcortical lesions was associated with a higher score on dimension 1 (active: *p* = 0.04; mixed: *p* = 0.02). Presence of CD20^+^ or CD138^+^ cells in the meninges was not associated with dimension 1 (CD20^+^: *p* = 0.70; CD138^+^: *p* = 0.70), as was presence of CD138^+^ cells in active and mixed lesions (active: *p* = 0.86; mixed: *p* = 0.89). Donors with a lesion in the brainstem tissue block that was used for investigating B cell presence scored higher on dimension 1 (*p* = 5.3 × 10^–7^), as expected (Fig. 3c) [13]. There was no significant association with the mean number of CD3^+^ cells in NAWM (normal-appearing white matter) of the pyramidal tract at the level of the medulla oblongata with dimension 1 (*p* = 0.54, rho = −0.09; suppl. Figure 14, Online Resource 1). However, CD3^+^ cell numbers in subcortical perilesional NAWM were positively correlated with dimension 1 (*p* = 0.04, rho = 0.39), as were CD3^+^ cell numbers in active but not mixed lesions (active: *p* = 0.08, rho = 0.50; mixed: *p* = 0.54, rho = 0.15) (Fig. 3d, e; suppl. Figure 15, Online Resource 1).

Dimension 1 correlated positively with axonal damage (NfL in CSF: *p* = 0.05, rho = 0.26); consistently, there seemed to be a lower axonal density and more axonal stress in donors with a high score on this dimension, although these associations were not significant (Bielschowsky: *p* = 0.48, rho = −0.10; APP^+^: *p* = 0.43) (suppl. Figure 16, Online Resource 1).

The PRS (polygenic risk score) and two individual variants (rs3135388, highly correlated with the HLA-DRB1*1501 allele [8], and rs10191329, the SNP that was recently associated with disease progression [23]) were not significantly associated with dimension 1 (PRS: *p* = 0.14, rho = 0.12; rs3135388: *p* = 0.17; rs10191329: *p* = 0.31) (Fig. 3f, g). However, the progression-associated SNP has a relatively low minor allele frequency and increased risk is mediated exclusively by the homozygous carriers. The five NBB donors homozygous for the risk allele (A) of rs10191329 (1.9 < dosage < 2.1) all scored high on dimension 1 (their median rank was 163, with the maximum possible rank being 194).

For a comprehensive overview of dimension 1, see Fig. 3h.

### Dimension 2: active lesions, ramified microglia, and an association with the HLA region

**Fig. 4:**
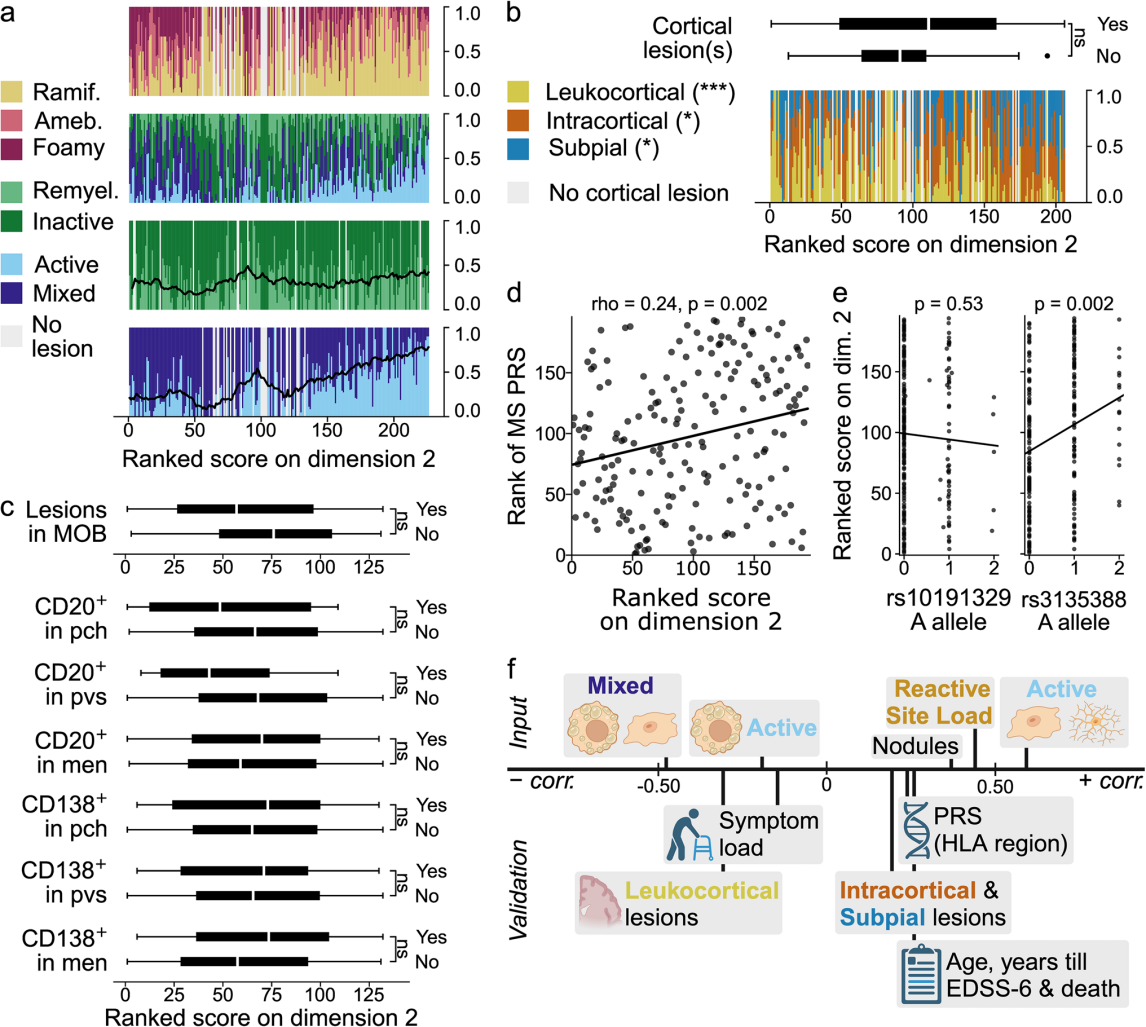
Dimension 2. Legends for **a–f** as for Figs. 3a–c, f–h, respectively; the only difference being that ranked scores of donors with and without CD20^+^ and CD138^+^ cells in subcortical lesions are not shown in **c** but in suppl. Figure 17 (Online Resource 1). HLA = human leukocyte antigen

Dimension 2 is associated with ramified microglia morphology and a corresponding lower microglial/macrophage activation score (suppl. Figure 12, Online Resource 1), as well as a shift from predominantly mixed to more active lesions. The ratio of remyelinated to inactive lesions remains quite stable (Fig. 4a).

Donors with (a) cortical lesion(s) did not score differently than those without (*p* = 0.38). There was a clear relation with cortical lesion location: scoring high on dimension 2 was associated with relatively fewer leukocortical lesions (*p* = 3.1 × 10^–4^, rho = -0.31), and more intracortical and subpial lesions (intracortical: *p* = 0.03, rho = 0.18; subpial: *p* = 0.02, rho = 0.21) (Fig. 4b & suppl. Figure 13, Online Resource 1).

There was no association between CD20^+^ and CD138^+^ cells in the brainstem and dimension 2 (for CD20^+^ cells: pch: *p* = 0.60; pvs: *p* = 0.12; meninges (men): *p* = 0.75; for CD138^+^ cells: pch: *p* = 0.82; pvs: *p* = 0.89; men: *p* = 0.29), and donors with or without a lesion in the brainstem did not score differently on the dimension (*p* = 0.19) (Fig. 4c). Presence of CD20^+^ and CD138^+^ cells in subcortical active and mixed lesions was also not associated with dimension 2 (for CD20^+^ cells: active: *p* = 0.94; mixed: *p* = 0.38; for CD138^+^ cells: active: *p* = 0.11; mixed: *p* = 0.86; suppl. Figure 17, Online Resource 1). Moreover, neither the mean number of CD3^+^ cells in NAWM nor in subcortical active and mixed lesions was correlated with dimension 2 (brainstem NAWM: *p* = 0.54, rho = −0.12; subcortical NAWM: *p* = 0.20, rho = 0.25; active: *p* = 0.20, rho = 0.35; mixed: *p* = 0.69, rho = 0.07; suppl. Figure 14 & 15, Online Resource 1).

Regarding axonal damage, density and stress, there was no correlation with dimension 2 (NfL in CSF: *p* = 0.73, rho = −0.04; Bielschowsky: *p* = 0.48, rho = −0.10; APP^+^: *p* = 0.99; suppl. Figure 16, Online Resource 1).

The PRS and the risk allele (A) of the HLA-DRB1*1501 tag SNP were associated with dimension 2 (PRS: *p* = 0.002, rho = 0.24; rs3135388: *p* = 0.002; Fig. 4d, e). After recalculation of the MS PRS without the HLA region, the PRS was no longer significantly correlated with dimension 2 (*p* = 0.37, rho = 0.09; suppl. Figure 18, Online Resource 1). Dimension 2 did not correlate with the progression SNP rs10191329 (*p* = 0.53; Fig. 4e), and did not show a particular clustering of the five homozygous carriers of the risk allele.

For an overview of dimension 2, see Fig. 4f.

### Dimension 3: loss of (white matter) lesion activity and scar formation

**Fig. 5:**
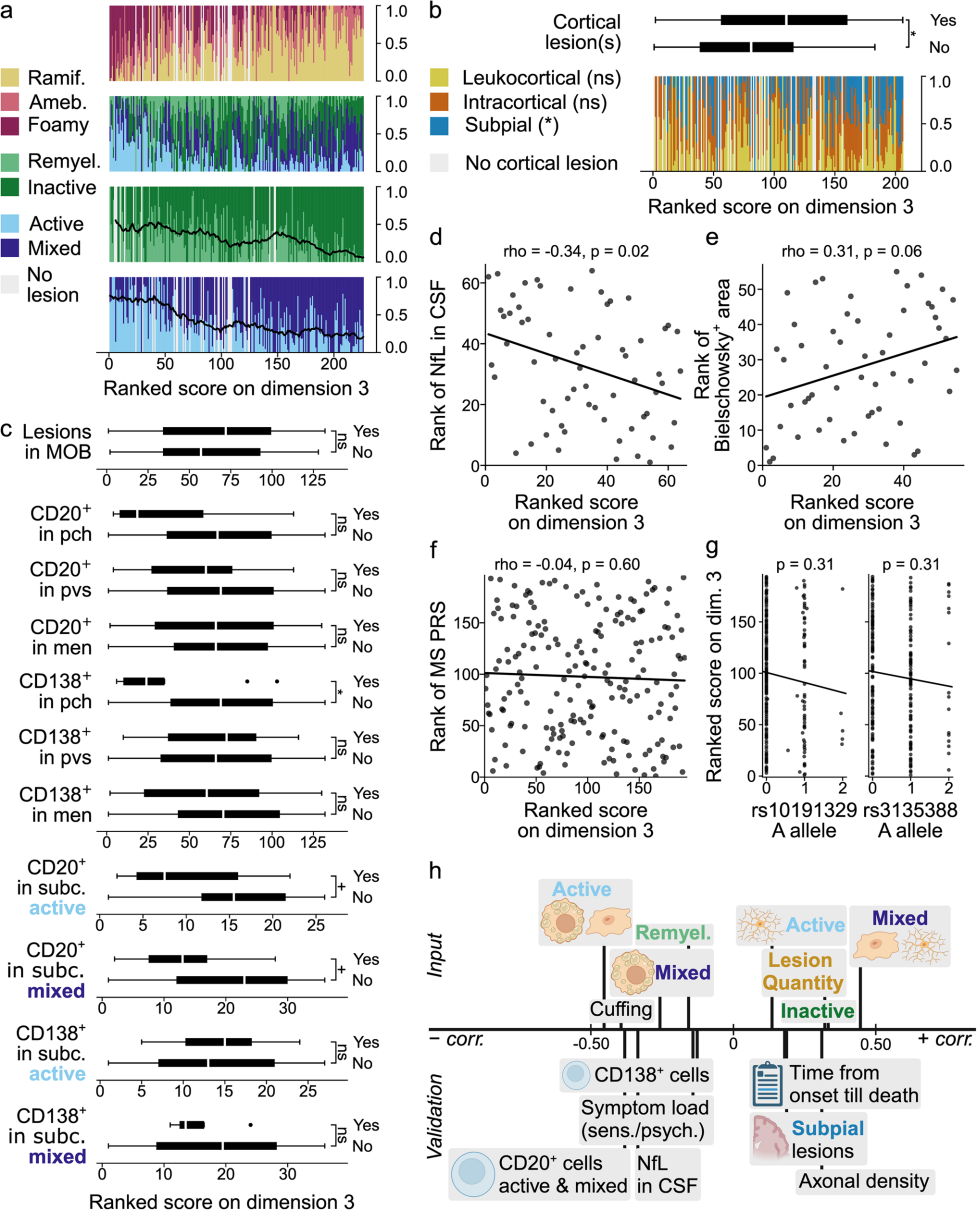
Dimension 3. Legends for **a–c** and **f–h** as for Fig. 3a–c, f–h, respectively. **d**, **e** Scatter plots with regression lines, depicting on the *Y*-axis the rank of CSF NfL levels for 64 donors (**d**) and the rank of the percentage Bielschowsky^+^ area for 55 donors (**e**), with donors ranked according to their score on dimension 3 on the *X*-axis. sens. = sensory/autonomic, psych. = psychiatric

Dimension 3 is negatively correlated with the microglial/macrophage activation score (suppl. Figure 12, Online Resource 1). In contrast to dimension 2, this was related to a relative increase of mixed compared to active lesions. Moreover, scoring higher on dimension 3 was associated with a decrease in the ratio of remyelinated to inactive lesions (Fig. 5a).

Donors with one or more lesions in the cortex generally scored higher on dimension 3 (*p* = 0.02). In addition, higher scoring donors had relatively more subpial lesions (*p* = 0.03, rho = 0.19). There was a non-significant negative correlation with intracortical and leukocortical lesion proportions (leukocortical: *p* = 0.11, rho = -0.14; intracortical: *p* = 0.65, rho = -0.03) (Fig. 5b & suppl. Figure 13, Online Resource 1).

Donors with CD138^+^ cells in the parenchyma but not the perivascular or meningeal regions of the brainstem scored lower on dimension 3 (pch: *p* = 0.04; pvs: *p* = 0.95; men: *p* = 0.18); donors with CD138^+^ cells in subcortical active and mixed lesions did not score differently (active: *p* = 0.89; mixed: *p* = 0.86). There was no significant association with the presence of CD20^+^ cells at any of the locations in the brainstem (pch: *p* = 0.17; pvs: *p* = 0.29; men: *p* = 0.70), or with the presence of a lesion in the investigated tissue block (*p* = 0.60). However, the presence of CD20^+^ cells in subcortical active and mixed lesions was associated with a lower score on dimension 3 (active: *p* = 0.08; mixed: *p* = 0.06) (Fig. 5c). Regarding T cells, there was no correlation between CD3^+^ cells in the NAWM or subcortical lesions and dimension 3 (brainstem NAWM: *p* = 0.88, rho = −0.02; subcortical NAWM: *p* = 0.51, rho = 0.13; active: *p* = 0.56, rho = -0.15; mixed: *p* = 0.20, rho = 0.29; suppl. Figure 14 & 15, Online Resource 1).

Dimension 3 was negatively correlated with axonal damage (NfL in CSF: *p* = 0.02, rho = −0.34) and positively with axonal density (Bielschowsky: *p* = 0.06, rho = 0.31) (Fig. 5d, e). Although the association with axonal stress was not significant, donors with APP^+^ axonal fragments or bulbs generally scored lower on dimension 3 (*p* = 0.42; suppl. Figure 16, Online Resource 1).

There was no significant association with the MS PRS (*p* = 0.60, rho = -0.04), the HLA-DRB1*1501 tag SNP (*p* = 0.31) or the progression SNP (*p* = 0.31) (Fig. 5f, g).

For an overview of dimension 3, see Fig. 5h.

## Discussion

Comprehension of pathophysiological processes contributing to MS is necessary to improve prognostic accuracy, therapeutic decision making, and development of disease-modifying therapies. Using a data-driven approach, we identified three biologically and clinically relevant dimensions of MS pathology. Our dimensions correspond to differences on the clinical and genetic level and are associated with grey matter lesional pathology, lymphocyte presence, and neuroaxonal damage. The three dimensions are likely associated with (1) immune cell activity and demyelination, (2) microglia (re)activity and possibly lesion initiation, and (3) loss of lesion activity and scar formation.

Our study highlights the importance of taking the heterogeneity within individual lesion types into account and considering (lesional) pathology in the broader context of a donor’s tendency towards certain neuropathological patterns, for which we offer a valuable tool in the form of scores on dimensions (Online Resource 3). It is both a strength and a limitation of our approach that our independent dimensions ‘overlap’ at the level of donors (i.e. each donor is characterised by its scores on all dimensions), because it helps with disentangling the heterogeneity of MS, while potentially complicating the comparison with and translation to findings on the donor level. Furthermore, it is important to recognize that the dimensions only explain part of the variation in the dataset, and the dataset itself is limited to mostly white matter neuropathology, with a focus on microglia. Grey matter pathology (independent of white matter pathology) and cell types such as oligodendrocytes and astrocytes fall outside the scope of the current study, and considering these in the future will further enhance our insight into MS.

Dimension 1 is positively associated with immune cell activity, demyelination, and clinical severity. Donors scoring low on dimension 1 had fewer lesions in white matter and cortex, a higher proportion of end-stage inactive and remyelinated lesions, and experienced a lower symptom load, longer time to EDSS-6, longer disease duration, and older age at death. Accordingly, prior research has associated extensive remyelination with an older age at death and a longer disease duration [46], and a low degree of cortical pathology with a milder disease course [4]. Conversely, donors scoring high on dimension 1 had more extensive pathology, which was associated with more severe disease. With regards to white matter, these donors were characterized by a predominance of active and mixed lesions, foamy microglia morphology, and the presence of nodules and cuffs. A previous post-mortem study of the NBB MS cohort had observed that clinical severity was positively correlated with lesion load and the proportion of mixed lesions [33]. Our study emphasizes the need to consider additional factors such as microglia morphology: having more lesions with foamy microglia (i.e. scoring high on dimension 1, or low on 2) is associated with more severe disease, whereas lesions with ramified microglia may not be as detrimental (i.e. scoring high on dimension 2 or 3). This could be due to axonal stress and acute axonal damage in lesions with foamy but not ramified microglia [58]; indeed, we found that dimension 1 correlated positively with axonal damage. Furthermore, the presence of B cells was associated with dimension 1, and not or negatively with dimension 2 and 3. In line with this, B cell presence has generally been related to clinically and pathologically more severe disease [13, 34, 42, 50]; in the NBB cohort, B cells have additionally been associated with the proportion of mixed lesions [13]. Perivascular B cells have previously been related to higher levels of T cells and microglia [42], fitting with the higher score on dimension 1 of donors with perivascular leukocyte cuffs and the positive correlation with CD3^+^ cell numbers in subcortical NAWM and active lesions. Moreover, all five homozygous risk allele carriers of the recently identified progression-associated SNP rs10191329, previously shown to be correlated with a shorter time to EDSS-6, higher lesion load, and higher cortical lesion rate in the NBB MS cohort [23], scored high on dimension 1. Taken together, these clinical, neuropathological, and genetic correlates consistently link dimension 1 with disease progression, and indicate that ongoing innate and adaptive immune cell activity and demyelination could be important therapeutic targets in progressive MS. Notably, as Bruton’s tyrosine kinase (BTK) is involved in both B cell and microglia activation, BTK inhibition may be a particularly promising therapeutic avenue [52, 57].

Dimension 2 is related to microglia (re)activity without demyelination and likely to lesion initiation. This dimension correlated positively with active lesions with ramified (and to a lesser extent ameboid) microglia, reactive sites, and microglia nodules. Interestingly, the latter two are both accumulations of ramified microglia in NAWM, accentuating the association between dimension 2 and ramified microglia morphology. Nodules are considered the first stage of lesion formation in MS and have previously been associated with active lesions [9, 59, 61], corresponding with our findings. Importantly, dimension 2 was associated with genetic risk in the HLA region, probably mainly due to an association with the HLA-DRB1*1501 risk allele. The HLA region and the broader MS PRS have previously been associated with the risk of developing MS [19, 47, 53], but have not been associated with the clinical severity during life [16, 22, 23, 53]. The negative correlation of dimension 2 with disease severity indicates that the HLA region is more relevant for lesion initiation, rather than expansion and progression of white matter lesions and disability in MS. Our data supports the idea that other factors, such as the vulnerability of white matter to tissue damage and adaptive immune activity, are important mediators of the latter endpoints. Shams et al. recently showed an association between the MS PRS and thalamic atrophy, while Yates et al. found that the HLA-DRB1*1501 risk allele correlated with neuropathology on autopsy, both indicating that genetic factors involved in disease onset also relate to end-organ injury [53, 63, 64]. Moreover, the PRS has been related to disease activity—specifically, the presence of relapses [53]. Since the pathology underlying clinical attacks are thought to be active lesions [12, 15], this seems to agree with our association of the HLA region with the specific neuropathological pattern of dimension 2.

Dimension 3 corresponds to a reduction in lesion activity and propensity for scar formation in relation to a protracted disease course. This dimension was positively associated with a higher lesion load and cortical lesion rate, more mixed compared to active and more inactive compared to remyelinated lesions, and ramified microglia morphology. This pattern correlated with a longer disease duration, without an obvious change in disease severity, and a higher age at onset of symptoms from the cognitive domain. Donors scoring high on dimension 3 had less axonal damage and a higher axonal density. Moreover, they were less likely to have cuffs, parenchymal plasma cells, and lesional B cells, in line with the remark by Fransen et al. that humoral involvement in white matter lesion activity may extinguish over time [13]. The lesional pattern of dimension 3 seems to partially reflect the finding of Frischer et al. [15], that active lesions become less frequent, while inactive, remyelinated, and smoldering (mixed) lesions more frequent with increasing disease duration. However, we found a decrease in the ratio of remyelinated to inactive lesions with an increasing score on dimension 3, which might be related to the lower remyelination capacity of mixed compared to active lesions [20].

Each dimension is associated with a distinct pattern of cortical pathology. In this autopsy cohort, mixed lesions have been associated with leukocortical and intracortical lesions (but not subpial lesions) [33]; an MRI study also noted this association between leukocortical and mixed (paramagnetic rim) lesions [1]. In line with this, dimension 2, which demonstrates a shift from a high proportion of mixed lesions towards a predominance of active lesions, negatively correlated with the leukocortical lesion proportion. Interestingly, both dimension 2 and 3 were positively correlated with the proportion of subpial lesions. This may be related to the decrease in the leukocortical lesion proportion for dimension 2, whereas it might reflect a primary increase in subpial lesion burden in donors scoring high on dimension 3, since these also have a higher cortical lesion rate. Demyelination from the meninges in the form of subpial lesions could be mechanistically different from demyelination in relation to perivascular grey and white matter lesions, and dimension 3 might mostly reflect the former. However, meningeal inflammation, subpial lesions, and disease progression are correlated [35], which is in apparent contrast with the longer disease duration and less frequent observations of cuffs, parenchymal plasma cells, and lesional B cells in donors scoring high on dimension 3. More insight in the relation between our dimensions, meningeal inflammation, and subpial lesions will require investigating the presence of meningeal B (and T) cells in regions other than the brainstem, distinguishing between diffuse lymphocyte infiltration and (B cell) follicles, and collecting more data on grey matter pathology.

We did not find an association between the dimensions and age at onset, sex, or clinical MS phenotype. The last supports the contemporary view that MS is one disease in which several types of events can occur (i.e. attacks and progression), rather than distinct clinical entities. Previously, donors with a documented relapsing disease were found to have a lower lesion load with relatively fewer mixed lesions and more remyelinated lesions compared to those with documented progressive disease in the NBB MS cohort [33], and donors with relapsing MS do seem to score somewhat lower on dimension 1 and 3. Clinically, females have a more benign disease course and a higher relapse rate, while males accumulate more disability [6, 36]. Neuropathologically, males have a higher proportion of mixed lesions and more often cortical pathology than females [15, 33]. Therefore, a difference between the sexes was expected. The current analysis, however, suggests that sex differences may become less apparent when considering neuropathological patterns in the form of dimensions instead of individual lesion types.

Our analyses of the relations between comorbidities and drug use and the neuropathological MS dimensions are correlational in nature, making it difficult to assess causality. Cardiovascular comorbidity and use of drugs to mitigate cardiovascular risk becomes more prevalent in populations when reaching an older age. This may (partially) explain the lower score on dimension 1 of donors with cardiovascular disease; a similar reasoning can be applied to the higher score on dimension 2 of donors with type 2 diabetes. Some autoimmune diseases are reported to be more common in MS patients, but the effects of comorbid autoimmunity on MS clinical severity and pathology and the underlying mechanisms are still largely unknown [43]. Further investigation on larger numbers of donors is needed before conclusions on the relation between autoimmune comorbidities and MS neuropathology can be drawn.

The dimensions could partially reflect historical changes, for instance related to the autopsy procedure (MRI-guided dissection was introduced in 2001), the availability of disease-modifying therapies, and the cohort itself (in the beginning of the NBB MS cohort, donors generally had more severe forms of MS). Since dimension 2 and 3 were positively correlated with year at autopsy, these two may be particularly affected by historical factors. The lack of an association between dimension 1 and year at autopsy seems to indicate that this dimension does not reflect the contemporary milder disease course of MS observed during the past decade [54]. In line with the development of MS disease-modifying therapies over the last decades, more recent autopsy cases were more frequently treated with disease-modifying therapies, and less often with MS-relevant immunosuppressant therapies such as prednisone pulses. In addition, we observed that donors who used drug therapies relevant for MS died at a younger age. This is in accordance with the higher score on dimension 1 for donors using disease-modifying drugs, and the lower score on dimension 2 for donors using MS-relevant immunosuppressants. Importantly, the direction of these associations makes confounding of our dimensions by MS treatment unlikely, and rather seems to validate them.

**Fig. 6:**
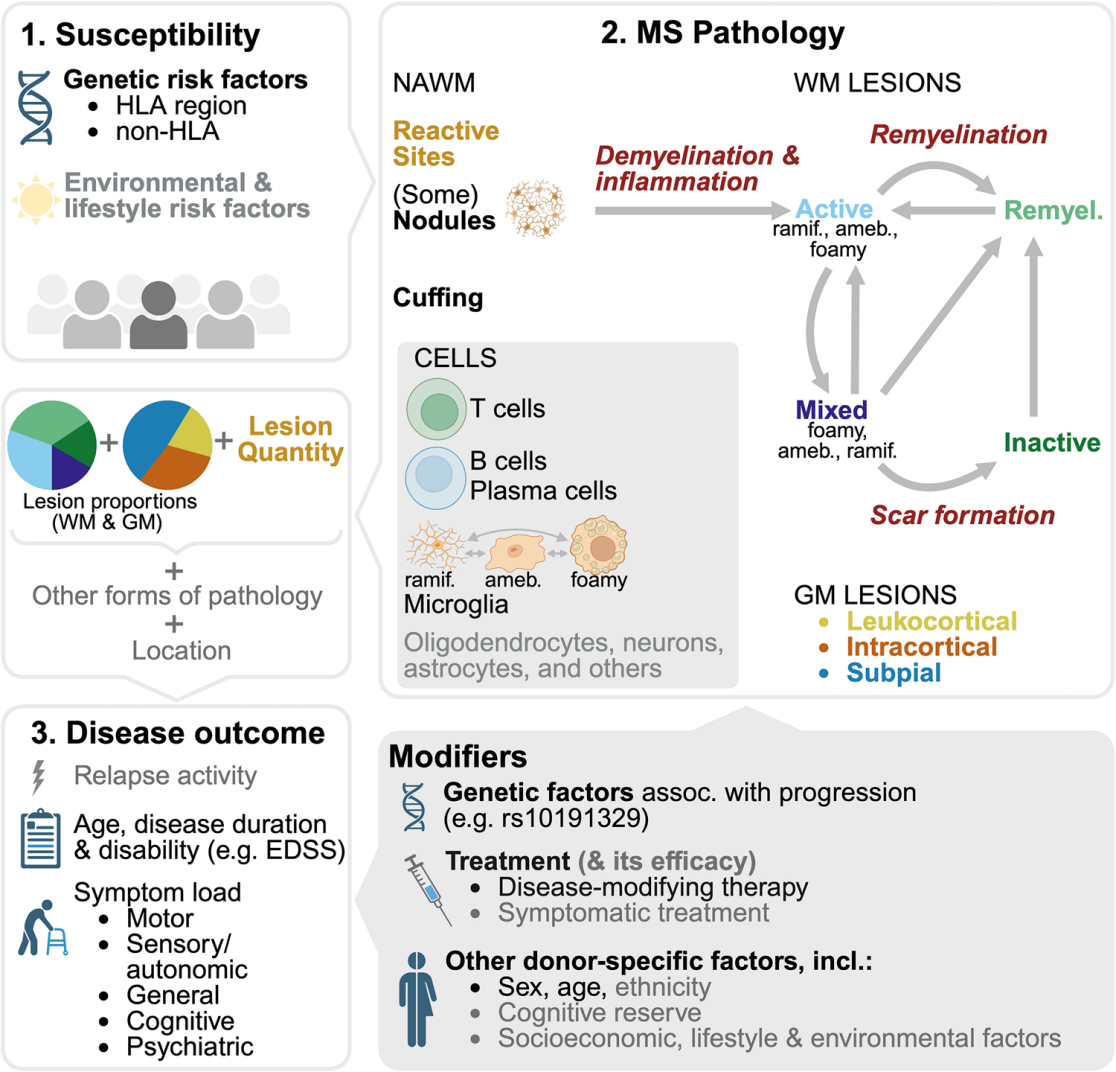
Overview of our (input and validation) variables in the larger conceptual framework of MS, created with BioRender.com. (1) MS development is driven by a combination of genetic, environmental, and lifestyle factors. Grey text refers to information not included in this study. (2) In white matter (WM), new active lesions form in the normal-appearing white matter (NAWM), possibly developing from a subset of microglia nodules, driven by demyelinating and inflammatory mechanisms. Several mechanisms (in dark red and italics) underlie the rates of WM lesion initiation, evolution and resolution. Immune and brain cells are involved in all steps. WM lesion pathology is correlated with pathology in grey matter (GM); independent (neurodegenerative) mechanisms contributing to GM lesion development are not shown. Lesion type and load, together with lesion location and other forms of pathology, largely determine clinical outcome. (3) Clinically, disease manifests in the form of relapses and disease progression, with the symptoms mainly depending on the brain and spinal cord regions that are affected. Several modifying genetic, therapeutic, and other donor-specific factors can influence MS pathology and/or clinical outcome. HLA = human leukocyte antigen; ramif. = ramified; ameb. = ameboid; EDSS = Expanded Disability Status Scale

In addition to different disease stages, age-related changes, and gradations in disease severity, our genetic analyses support the idea that (some of) the dimensions may partially represent different mechanisms that contribute to disease evolution in individuals with MS. This is in accordance with the view of Kuhlmann et al. [28], who suggest that instead of one disease mechanism underlying MS, there is a combination of mechanisms of injury and repair, with varying importance between patients and over time. Conceptual Fig. 6 illustrates how the variables in our study—and thereby the dimensions—fit within the broader context of MS.

In conclusion, we identified three dimensions related to initiation and progression of MS. Knowledge of donors’ scores on these dimensions—and what this represents—will aid donor selection in future studies that aim to investigate the distinct underlying mechanisms of MS and thereby disentangle the heterogeneity. Ultimately, achieving stratification of MS patients based on pathobiology in research and clinical settings will require a firm link between these neuropathological mechanisms and biomarkers—including but not limited to genetic and imaging factors.

### Supplementary Information

Below is the link to the electronic supplementary material.

Online Resource 1:.pdf file with Suppl. Figures 1–18. (PDF 19313 KB)

Online Resource 2:.xlsx file with raw input and anonymised validation data (XLSX 66 KB)

Online Resource 3:.xlsx file with (ranked & unranked) scores of NBB MS donors on the dimensions. (XLSX 27 KB)

## Data Availability

The majority of the datasets used and analysed in the current study are provided as supplementary material (Online Resource 2 & 3); clinical disease trajectories of NBB donors are accessible via the website of the NND (Netherlands Neurogenomics Database; https://nnd.app.rug.nl). Anonymisation was performed by adjusting age at onset and death as well as time from onset to EDSS-6 and death to 5-year intervals, with donors aged < 36 years grouped into the ‘ < 36’ category. Information regarding cause of death categories with < 10 donors (i.e. suicide) was not provided. Original data and data involving year of death, clinical diagnosis, and drug use are available upon reasonable request, by contacting the NBB (eNBB@nin.knaw.nl) or its director, I. Huitinga (i.huitinga@nin.knaw.nl). Note that the identifiers for NBB MS donors in Online Resource 2 & 3 correspond to those on the NND website.
