# Supplementary material for: Disentangling the heterogeneity of multiple sclerosis through identification of independent neuropathological dimensions
Source: Acta Neuropathol. 2024 May 21;147(1):90. doi: 10.1007/s00401-024-02742-w (PMC11108935; doi:10.1007/s00401-024-02742-w)
Supplement: Supplementary file 1 — Online Resource 1:.pdf file with Suppl. Figures 1–18. (PDF 19313 KB) [file 401_2024_2742_MOESM1_ESM.pdf]

Online Resource 1: Suppl. Figures 1-18

Article title: Disentangling the heterogeneity of multiple sclerosis through identification of independent neuropathological dimensions

Journal name: Acta Neuropathologica

Author names: Alyse de Boer, Aletta M.R. van den Bosch, Nienke J. Mekkes, Nina Fransen, Ekaterina Dagkesamanskaia, Eric Hoekstra, Jörg Hamann, Joost Smolders, Inge Huitinga, Inge R. Holtman

Corresponding author: Inge R. Holtman
Section Molecular Neurobiology, Department of Biomedical Sciences, University Medical Center Groningen, University of Groningen, Groningen, The Netherlands
The Netherlands Brain Bank, Netherlands Institute for Neuroscience, Amsterdam, The Netherlands
i.r.holtman@umcg.nl

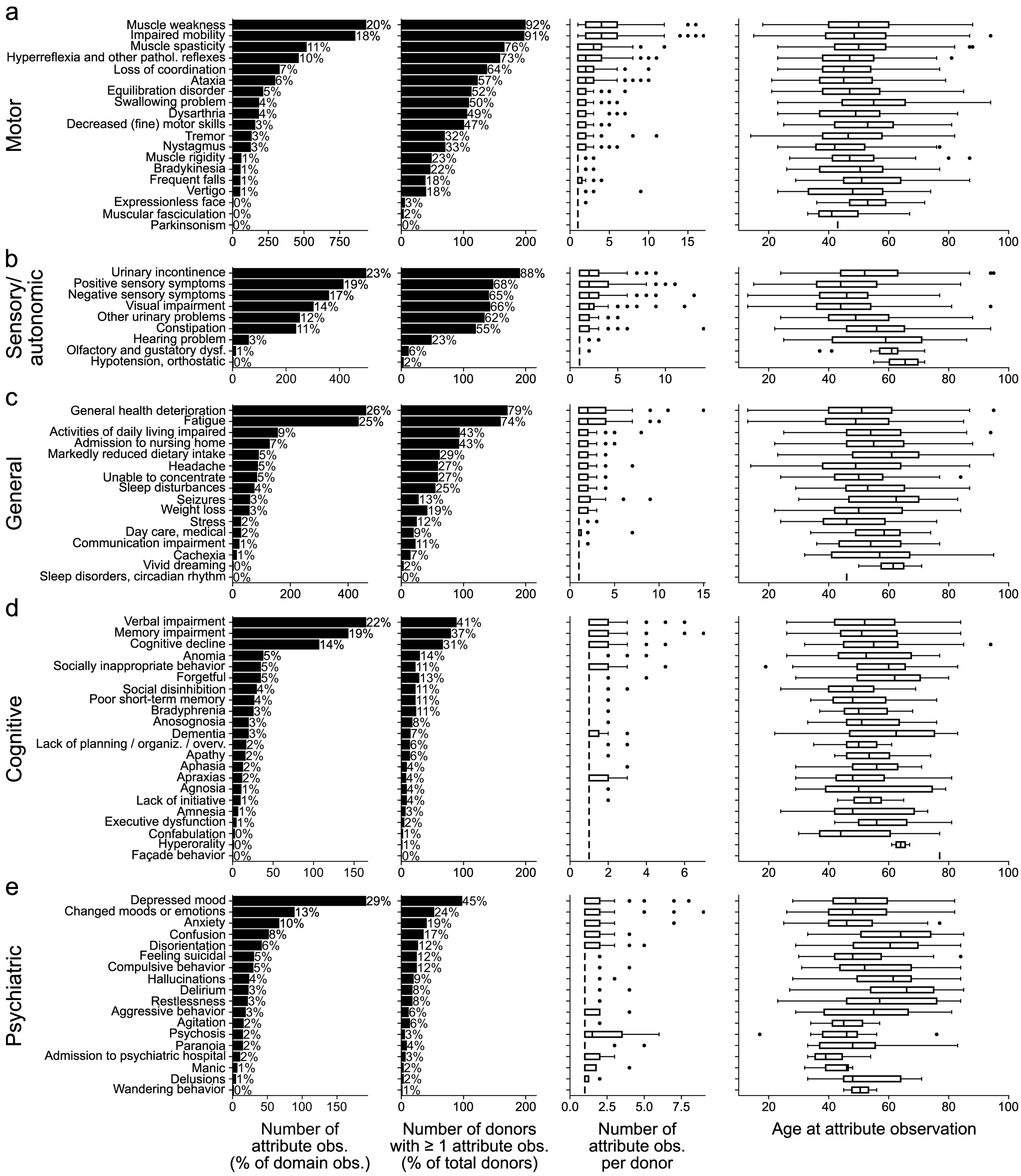

Suppl. Fig. 1 An overview of the clinical history of 217 MS donors, with each row **a-e** depicting from left to right 1) a horizontal bar graph showing how often the attributes of the domain were observed, labelled with the percentages relative to the total number of domain observations, 2) a horizontal bar graph showing how many donors experienced the attributes at least once, labelled with percentages relative to the total number of MS donors in the cohort for which clinical history was available (n = 217), 3) box plots showing how often an attribute was observed in the lifetime of donors who experienced the attribute at least once, and 4) box plots showing the ages at which the attribute was observed. Note that X-axis scale differs among the different plots. Also note that the resolution for attribute observations is one year. Sometimes, the year of/age at observation was unknown; these observations are included in all plots except the box plots displaying the age distribution of attribute observation (4). **a** In total, there were 4654 observations of motor domain attributes in 213 donors. Of these, age at observation was known for 4644. **b** Age was known for 2138 of 2147 sensory/autonomic domain observations in 214 donors. **c** Age was known for 1745 of 1750 general domain observations

in 211 donors. **d** Age was known for 741 of 743 cognitive domain observations in 160 donors. **e** Age was known for 659 of 662 psychiatric domain observations in 153 donors. pathol. = pathological; dysf. = dysfunction; organiz. = organization; overv. = overview; obs. = observation(s)

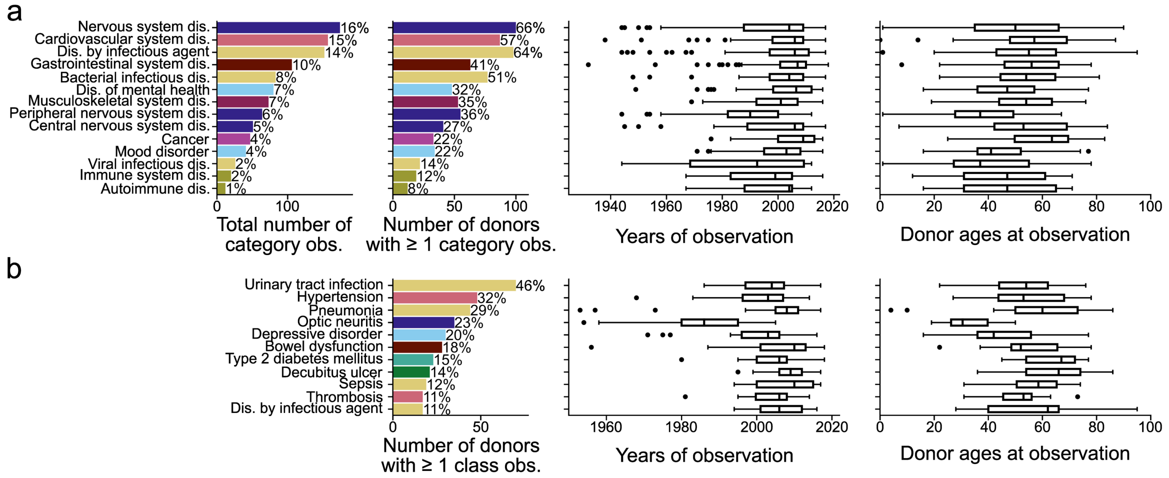

Suppl. Fig. 2 An overview of the comorbidities in the MS cohort. Clinical diagnosis data was available for 215 MS donors; 317 unique Netherlands Neurogenomics Database Human Disease Ontology (NND-HDO) classes were observed. For more details on the classes and the ontological structure of the NND-HDO, see https://nnd.app.rug.nl or BioPortal (https://bioportal.bioontology.org/ontologies/NND_CD). The word ‘category’ is used to refer to a higher-level class together with its subclasses, while ‘class’ indicates only the class itself. If a clinical diagnosis was not included in the NND-HDO, the most appropriate higher-level class was assigned. Moreover, the certainty of the clinical diagnoses could not be determined; a class observation indicates that either the class itself, or a diagnosis falling within the class, was at one point considered in the clinical diagnostic process. Donors with no observations of classes other than MS or its subclasses were not considered in our analysis; if a class was observed multiple times for a donor, only the class observation at the earliest time point was included. In total, 1074 observations in 152 donors remained; of these 1074 observations, the year was known for 953 and age for 912 observations. In addition to 14 relevant categories (**a**), the 11 most commonly observed classes were analysed (**b**). In **a**, from left to right 1) a horizontal bar graph showing how often the categories were observed (only considering the unique classes falling within that category), labelled with the percentages relative to the total number of class observations (n = 1074), 2) a horizontal bar graph showing in how many donors the category was observed at least once, labelled with percentages relative to the total number of donors in the cohort with an observation other than MS (n = 152), 3) box plots showing the years in which the category was observed, and 4) box plots showing the ages at which the category was observed. In **b** similar graphs as those in 2), 3) and 4) of **a**, but than for classes. Note that X-axis scale differs among the different plots. Categories or classes in **a** and **b** with the same colour are subclasses of a higher-level category included in the analysis. For example, the categories ‘peripheral nervous system disease’ and ‘central nervous system disease’ are both subclasses of ‘nervous system disease’, and ‘optic neuritis’ is a class within ‘peripheral nervous system disease’. Note that overlap between categories is common. For instance, ‘autoimmune disease of the nervous system’ is a subclass of ‘nervous system disease’ as well as ‘autoimmune disease’. Age at diagnosis was calculated as age at death – (year of death – year at diagnosis) and may slightly deviate from a donor’s true age at diagnosis (depending on the date of birth). dis = disease; obs. = observation

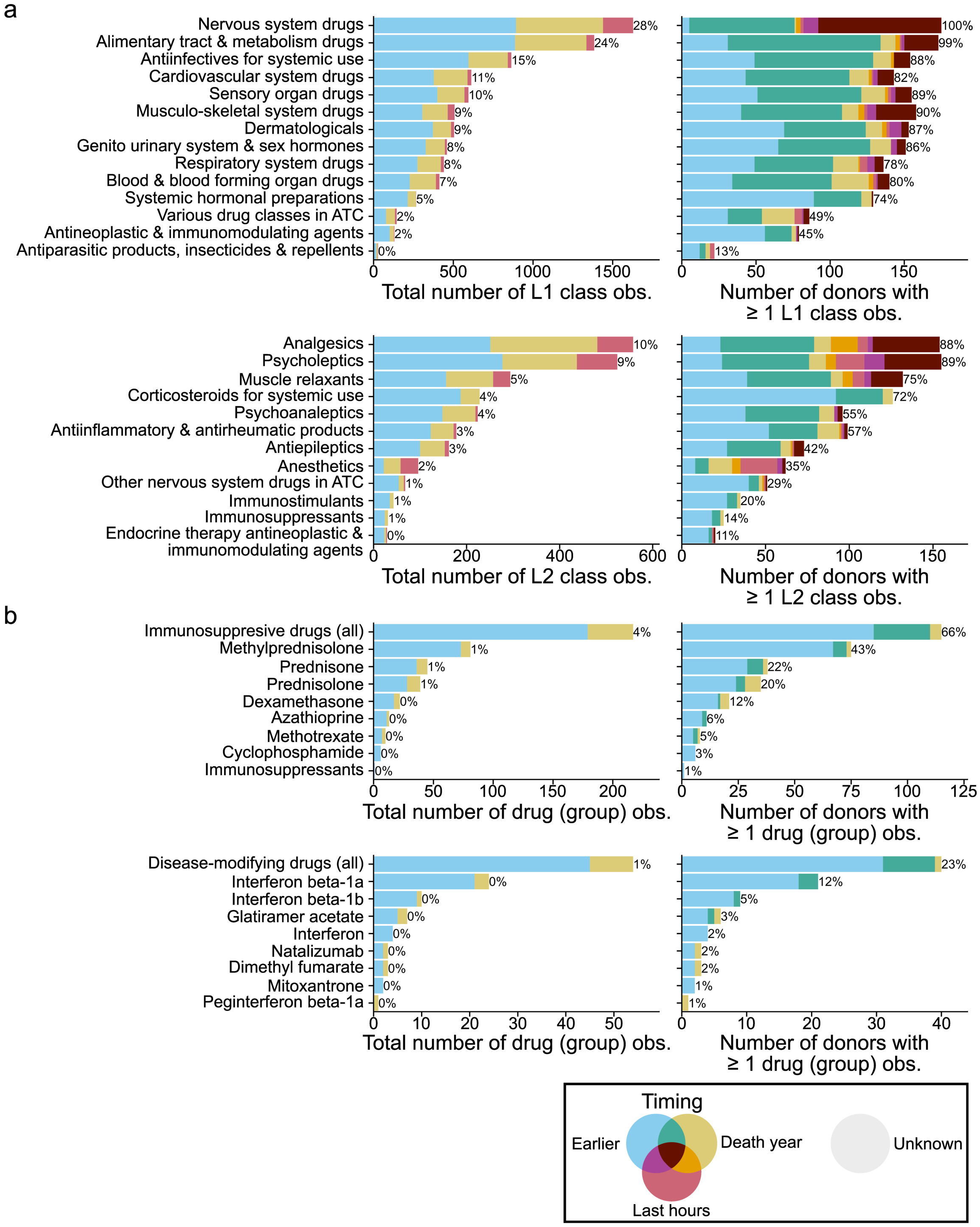

Suppl. Fig. 3 An overview of drug use in the MS cohort. Medication data was available for 217 MS donors. Drug names were extracted from the donor files using text-parsers, preprocessed, and matched to Anatomical Therapeutic Chemical (ATC) codes; 604 unique ATC classes were observed. Timepoints were categorised into 'last hours', 'death year', 'earlier', and 'unknown', and ATC codes were binarized at the level of these time categories. Note that if timepoints were not identified by the parser, drug text was assigned to the following timepoint (e.g. drugs observed for the 'last 24 hours' would then be assigned to 'last year'). Donor files with ≥ 200 characters and ≥ 25 words in the medication summary that contained observations in both the year of death (i.e. either 'last hours' or 'death year') and another year (i.e. 'earlier' or 'unknown') were included in the analysis; 5830 observations of 585 unique ATC classes in 175 donors remained. To assess drug treatment relevant for MS, a list was composed with 1) the most commonly observed drugs (over all time categories), 2) the

drugs most commonly used by the donors, 3) all drugs within the L1 class 'NERVOUS SYSTEM DRUGS', and 4) all drugs within the L1 class 'ANTINEOPLASTIC AND IMMUNOMODULATING AGENTS'. For each drug on this list, we determined if it was a disease-modifying or immunosuppressive drug prescribed for MS. In **a**, from left to right 1) a horizontal bar graph showing how often the ATC classes were observed, coloured per time category, and labelled with the percentages relative to the total number of observations (n = 5830), and 2) a horizontal bar graph showing in how many donors the classes were observed at least once, coloured for the (combination of) timepoints, and labelled with percentages relative to the total number of donors considered in the analysis (n = 175), for all L1 (top) and a subset of L2 (bottom) classes. In **b** similar graphs as 1) and 2) in **a**, but than for immunosuppressive drugs (top) and disease-modifying drugs (bottom) relevant for MS. 'Immunosuppressive drugs (all)' (top row) shows the total number of observations of this drug group (i.e. the sum of the number of observations of the drugs listed in the rows below) (left), and the number of donors with at least one observation of a drug in this group (right); idem for 'Disease-modifying drugs (all)'. Note that X-axis scale differs among the different plots. The class and drug names in **a** and **b** may slightly deviate from the preferred ATC names (regarding the use of capital letters and ampersands). The L1 class 'SYSTEMIC HORMONAL PREPARATIONS, EXCL. SEX HORMONES AND INSULINS' was abbreviated to 'Systemic hormonal preparations'. obs. = observation

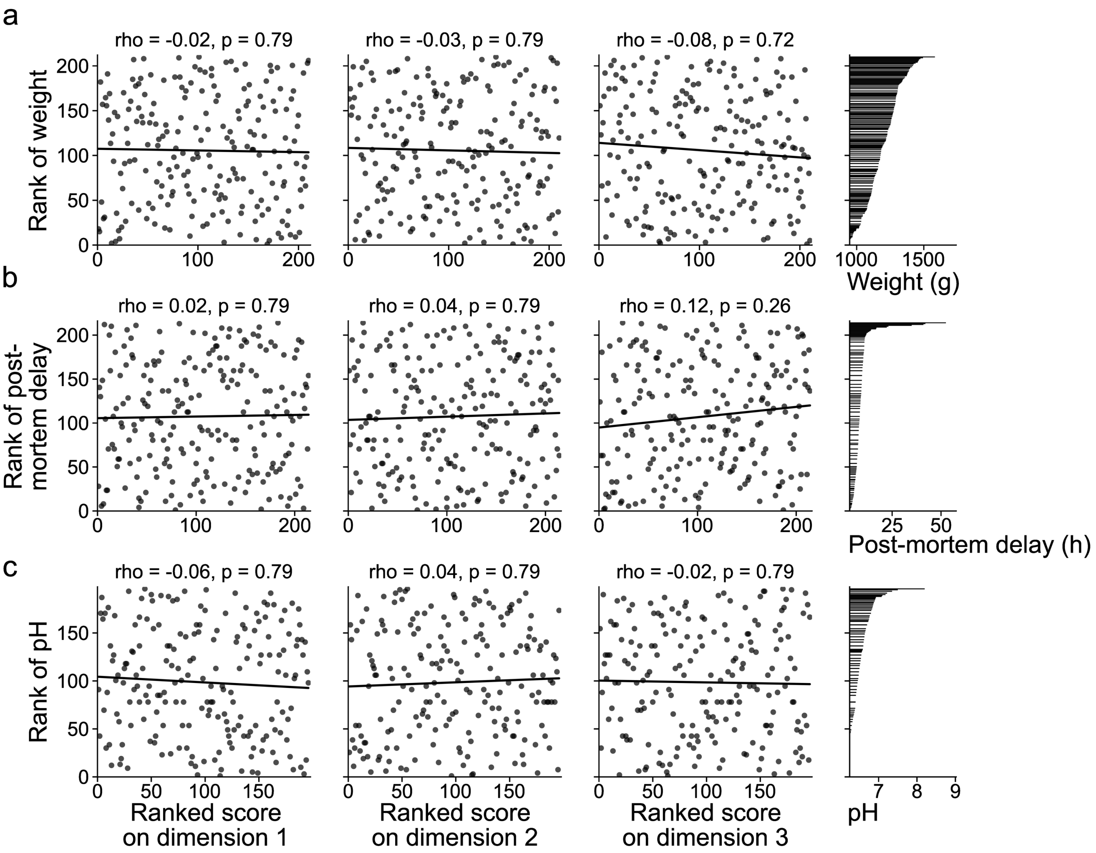

Suppl. Fig. 4 Association between technical covariates and dimensions. Note that X-axis scale differs among the different subplots. **a-c** Scatter plots with regression lines, depicting on the Y-axis the rank of brain weight for 210 donors (**a**), post-mortem delay for 214 donors (**b**), and pH for 196 donors (**c**), respectively, with donors ranked according to their score on dimension 1-3 on the X-axes. The bar plots depict the unranked value on the X-axis per rank on the Y-axis; brain weight is indicated in grams (g) and post-mortem delay in hours (h). Significance was assessed with Spearman correlation and FDR-adjusted for multiple testing. Ties were assigned averaged ranks. FDR = False Discovery Rate

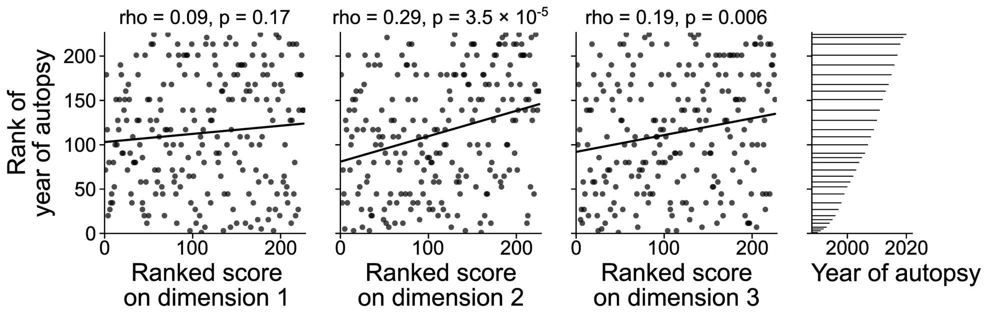

Suppl. Fig. 5 Association between year of autopsy and dimensions. Scatter plots with regression lines show the rank of year of autopsy on the Y-axis for all 226 donors, with donors ranked according to their score on dimension 1-3 on the X-axes. The bar plot depicts the unranked value on the X-axis per rank on the Y-axis. Significance was assessed with Spearman correlation and FDR-adjusted for multiple testing. Ties were assigned average ranks. FDR = False Discovery Rate

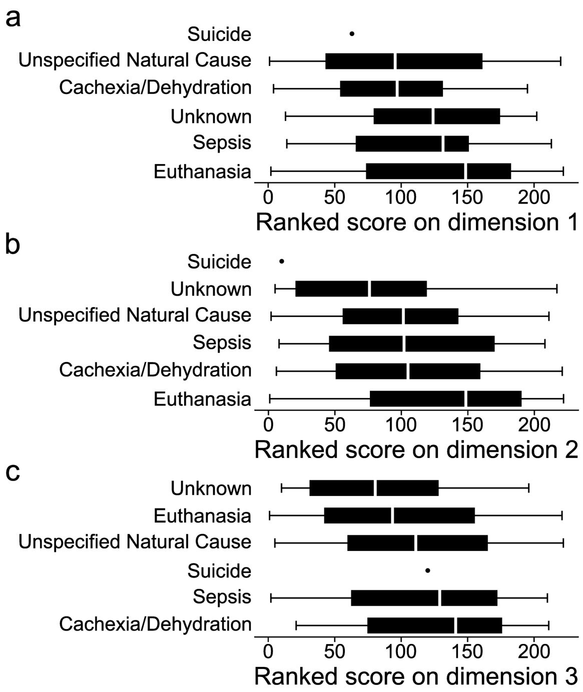

Suppl. Fig. 6 Association between cause of death (CoD) and dimensions. Box plots showing the ranked scores of donors, per dimension, per CoD. CoD was known for 222 donors (1 suicide, 62 euthanasia, 22 sepsis, 23 cachexia/dehydration, 104 unspecified natural cause, 10 unknown). There were no significant differences between the different causes for dimension 1 and 3 (Kruskal-Wallis; dim. 1: $p = 0.12$, 2: $p = 0.05$, 3: $p = 0.51$). Donors who died by euthanasia scored significantly higher on dimension 2 than donors who died because of unspecified natural causes (Mann-Whitney U; $p = 0.02$). Significance was FDR-adjusted for multiple testing. Ties were assigned average ranks. FDR = False Discovery Rate

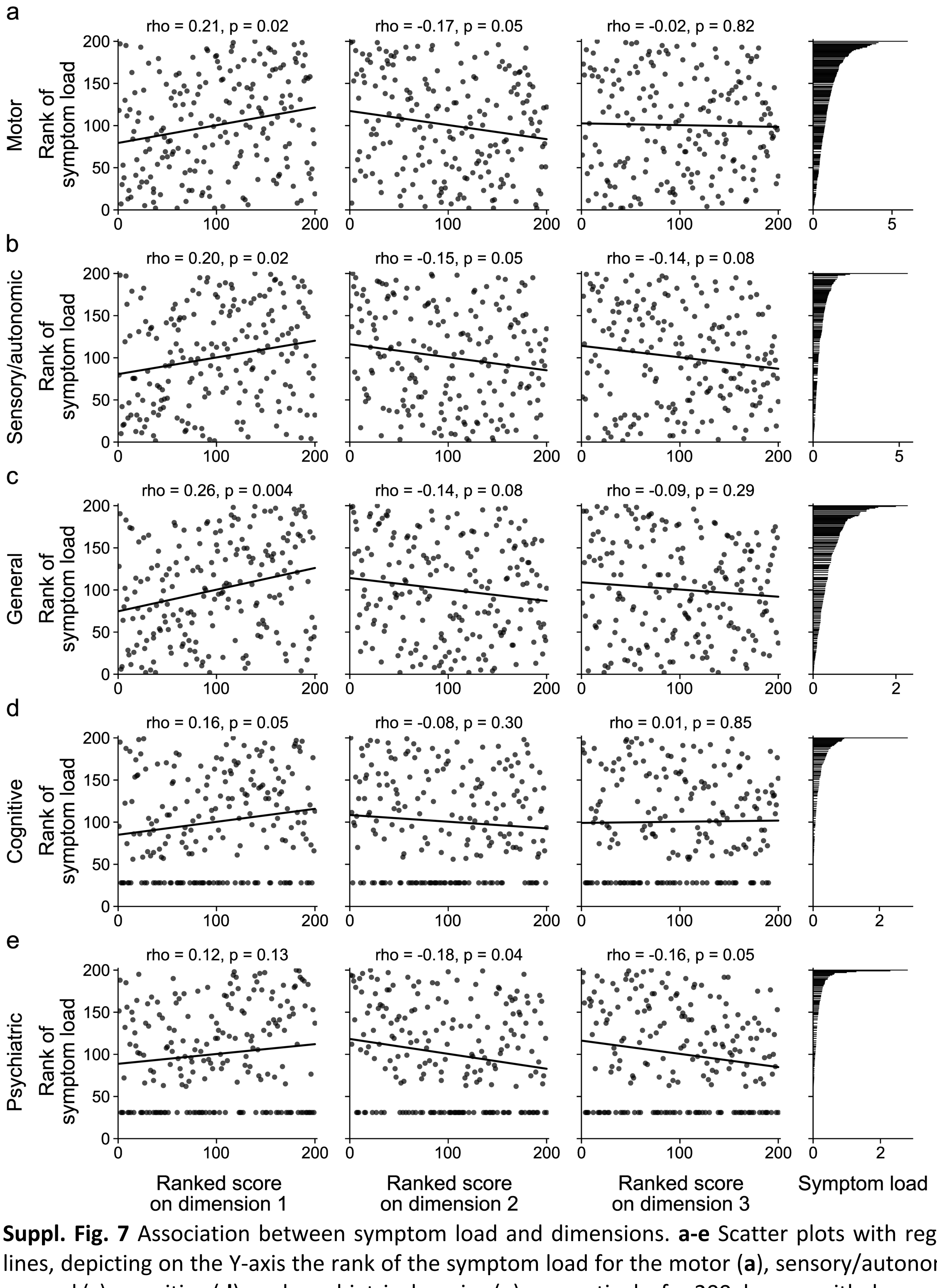

Suppl. Fig. 7 Association between symptom load and dimensions. **a-e** Scatter plots with regression lines, depicting on the Y-axis the rank of the symptom load for the motor (**a**), sensory/autonomic (**b**), general (**c**), cognitive (**d**), and psychiatric domains (**e**), respectively, for 200 donors, with donors ranked according to their score on dimension 1-3 on the X-axes. The bar plots depict the unranked value on the X-axis per rank on the Y-axis. Symptom load is defined as the total number of signs and symptoms adjusted for disease duration. Significance was assessed with Spearman correlation and FDR-adjusted for multiple testing. Ties were assigned averaged ranks. FDR = False Discovery Rate

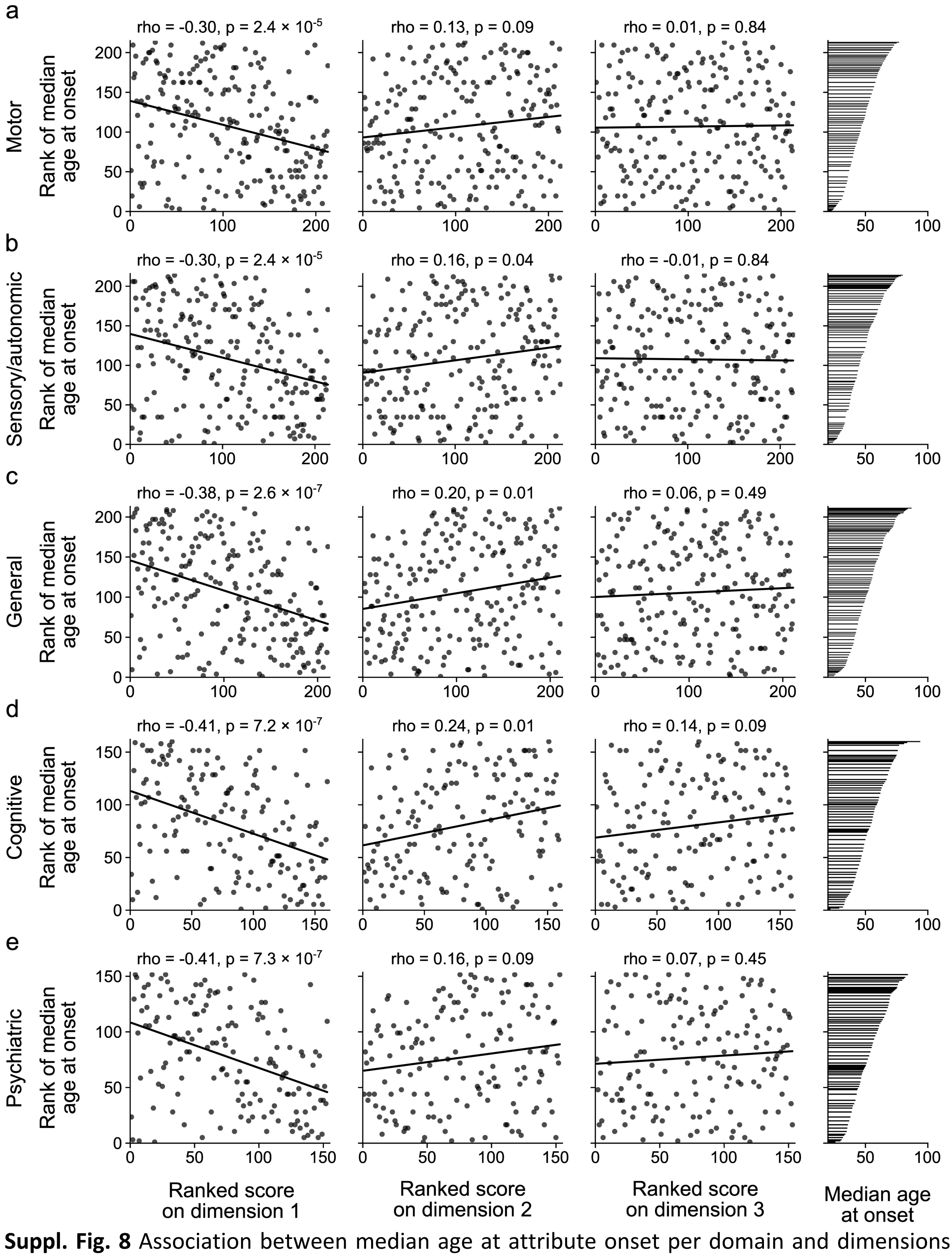

Suppl. Fig. 8 Association between median age at attribute onset per domain and dimensions. Note that X-axis scale differs among the different subplots. **a-e** Scatter plots with regression lines, depicting on the Y-axis the rank of the median age at onset for attributes of the motor domain for 213 donors (**a**), of the sensory/autonomic domain for 214 donors (**b**), of the general domain for 211 donors (**c**), of the cognitive domain for 160 donors (**d**), and of the psychiatric domain for 153 donors (**e**), respectively, with donors ranked according to their score on dimension 1-3 on the X-axes. The bar plots depict the unranked value on the X-axis per rank on the Y-axis. The median age at onset was determined by identifying all ages at which the attributes within a domain were first observed and taking the median value. Significance was assessed with Spearman correlation and FDR-adjusted for multiple testing. Ties were assigned averaged ranks. FDR = False Discovery Rate

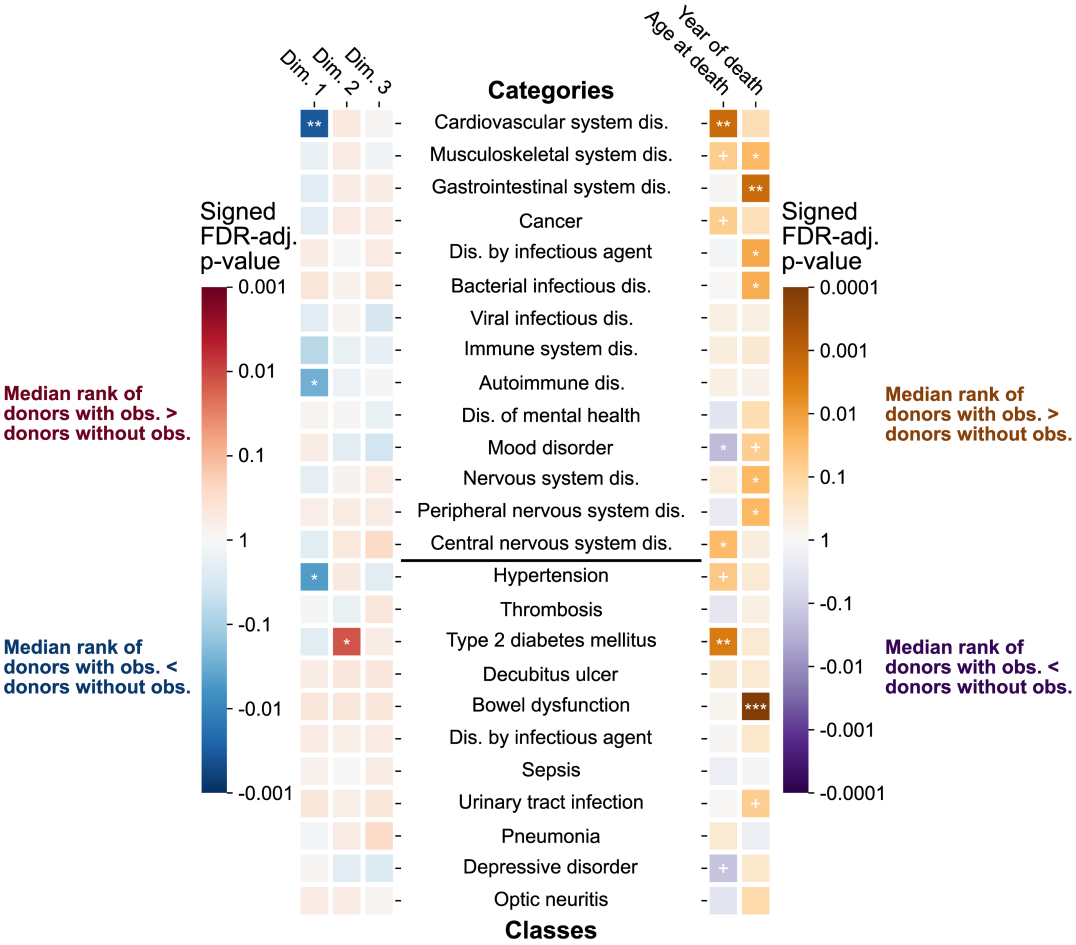

Suppl. Fig. 9 Heatmaps of the relation between comorbidities and dimensions, age at death, and year of death. Of donors with a class observation other than MS or its subclasses (n = 152), the scores on dimensions 1-3 (left) and the age at death and year of death (right) of donors with and without one or more category or class observations were compared. Age at death was known for 145 donors. Significance was assessed with Mann-Whitney U; FDR-adjustment for multiple testing was performed for all comparisons involving both categories and classes and dimensions 1-3 (left), and separately for all comparisons involving both categories and classes and age at death and year of death (right). Significant associations are marked with a symbol. The black line divides the labels of the heatmap between categories (top) and classes (bottom). The colour in the heatmap reflects the negative log10 of the FDR-adjusted p-value, which was signed to reflect the direction of the effect: red or orange indicates that the median rank of donors with at least one category or class observation was higher than the median rank of donors without an observation, blue or purple the opposite. Thus, donors with at least one observation of the category 'cardiovascular system diseases' scored lower on dimension 1 and died at an older age. FDR = False Discovery Rate dis = disease; dim. = dimension; obs. = observation; + p ≤ 0.1; * p ≤ 0.05; ** p ≤ 0.01; *** p ≤ 0.001; **** p ≤ 0.0001

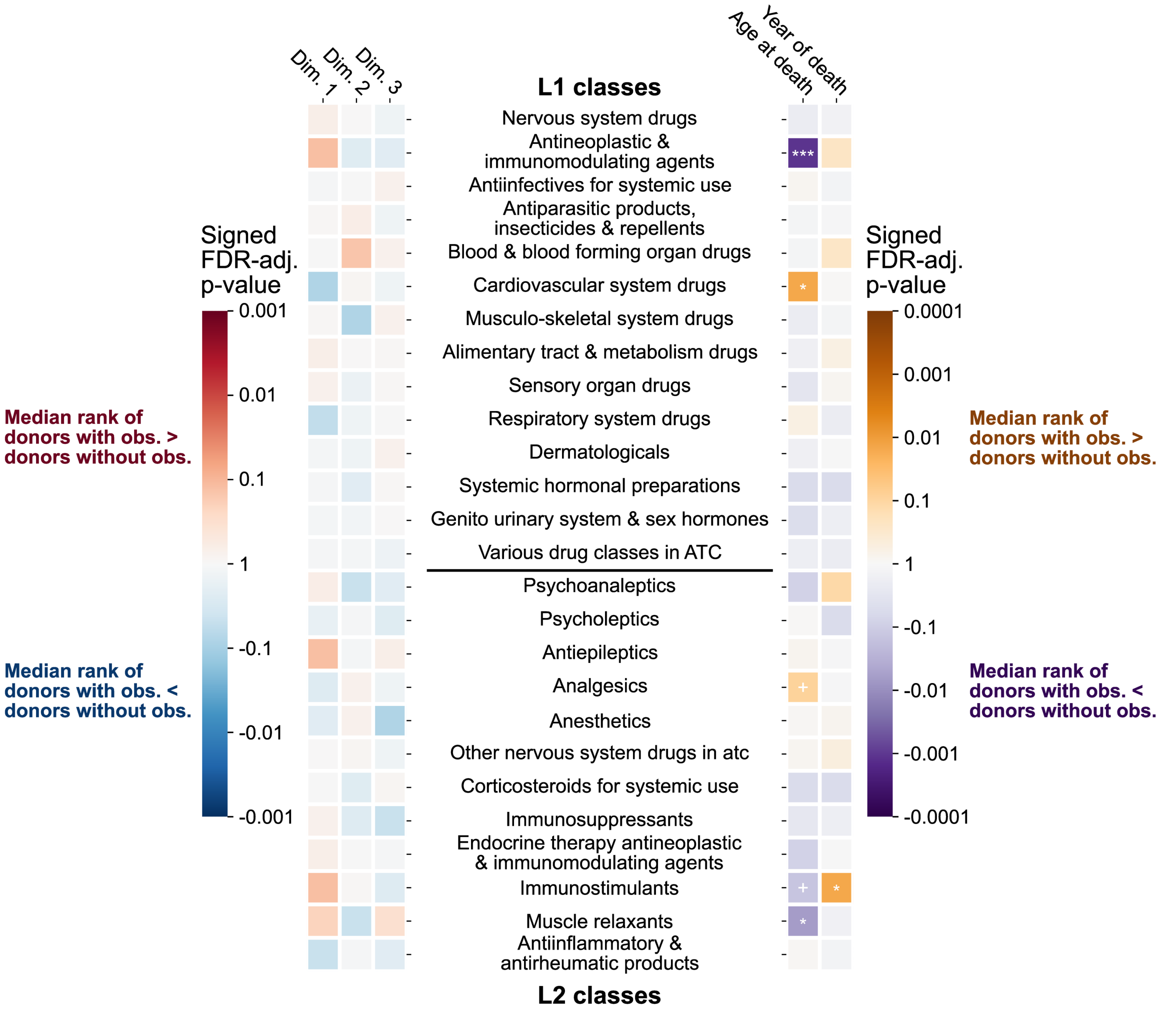

Suppl. Fig. 10 Heatmaps of the relation between Anatomical Therapeutic Chemical (ATC) level 1 (L1) and level 2 (L2) drug classes and dimensions, age at death, and year of death. Of 175 MS donors with a high-quality medication section in the donor file, the scores on dimensions 1-3 (left) and the age at death and year of death (right) of donors with and without one or more class observations were compared. Age at death was known for 166 donors. Significance was assessed with Mann-Whitney U; FDR-adjustment for multiple testing was performed for all comparisons involving both L1 and L2 classes and dimensions 1-3 (left), and separately for all comparisons involving both L1 and L2 classes and age at death and year of death (right). Significant associations are marked with a symbol. The black line divides the labels of the heatmap between L1 (top) and L2 classes (bottom). The colour in the heatmap reflects the negative log10 of the FDR-adjusted p-value, which was signed to reflect the direction of the effect: red or orange indicates that the median rank of donors with at least one L1 or L2 class observation was higher than the median rank of donors without an observation, blue or purple the opposite. Thus, donors with at least one observation of the class 'Antineoplastic & immunomodulating agents' died at a younger age. The class names may slightly deviate from the preferred ATC names (regarding the use of capital letters and ampersands); the L1 class 'SYSTEMIC HORMONAL PREPARATIONS, EXCL. SEX HORMONES AND INSULINS' was abbreviated to 'Systemic hormonal preparations'. FDR = False Discovery Rate; dim. = dimension; obs. = observation; + $p \leq 0.1$; * $p \leq 0.05$; ** $p \leq 0.01$; *** $p \leq 0.001$; **** $p \leq 0.0001$

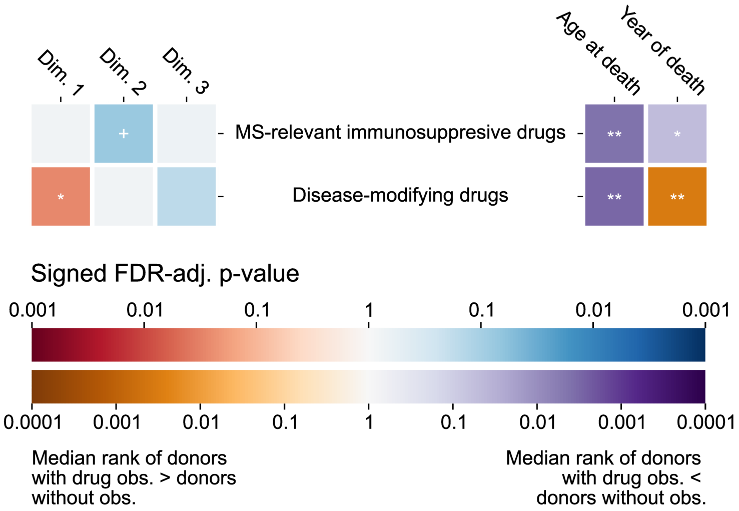

Suppl. Fig. 11 Heatmaps of the relation between MS-relevant drug therapies and dimensions, age at death, and year of death. Of 175 MS donors with a high-quality medication section in the donor file, the scores on dimensions 1-3 (left) and the age at death and year of death (right) of donors with and without one or more observations of relevant immunosuppressive or disease-modifying drugs were compared. Age at death was known for 166 donors. Significance was assessed with Mann-Whitney U; FDR-adjustment for multiple testing was performed for all comparisons involving dimensions 1-3 (left), and separately for all comparisons involving age at death and year of death (right). Significant associations are marked with a symbol. The colour in the heatmap reflects the negative log10 of the FDR-adjusted p-value, which was signed to reflect the direction of the effect: red or orange indicates that the median rank of donors with at least one drug observation was higher than the median rank of donors without an observation, blue or purple the opposite. FDR = False Discovery Rate; dim. = dimension; obs. = observation; + p ≤ 0.1; * p ≤ 0.05; ** p ≤ 0.01; *** p ≤ 0.001; **** p ≤ 0.0001

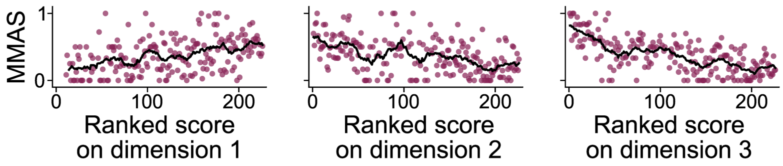

Suppl. Fig. 12 Association between microglia/macrophage activation score (MMAS) and dimensions. Scatter plots with fitted lines, depicting the values for the MMAS on the Y-axis, for 226 donors ranked according to their score on dimension 1-3 on the X-axes. In case of ties, donors were assigned ranks in the order of appearance in the dataset, so that each donor received a unique rank. The line represents the centered moving average, over a window of 20 observations, with a maximum of 10 missing values. Note that MMAS was only determined for donors with active and/or mixed lesions (202/226 donors)

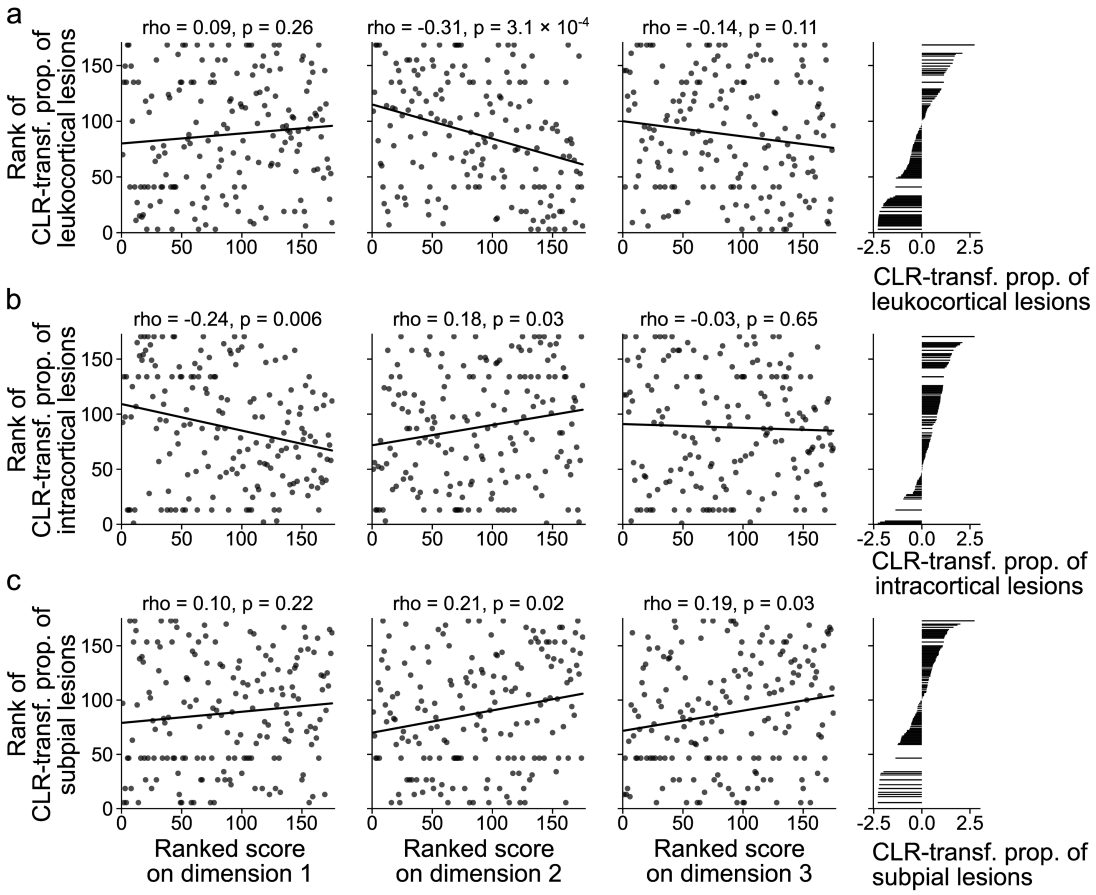

Suppl. Fig. 13 Association between cortical lesion types and dimensions. **a-c** Scatter plots with regression lines, depicting on the Y-axis the rank of CLR-transformed proportions (transf. prop.) of leukocortical lesions (**a**), intracortical lesions (**b**), and subpial lesions (**c**), respectively, for 175 donors, with donors ranked according to their score on dimension 1-3 on the X-axes. The bar plots depict the unranked value on the X-axis, per rank on the Y-axis. Significance was assessed with Spearman correlation and FDR-adjusted for multiple testing. Ties were assigned averaged ranks. CLR = centered log ratio; FDR = False Discovery Rate

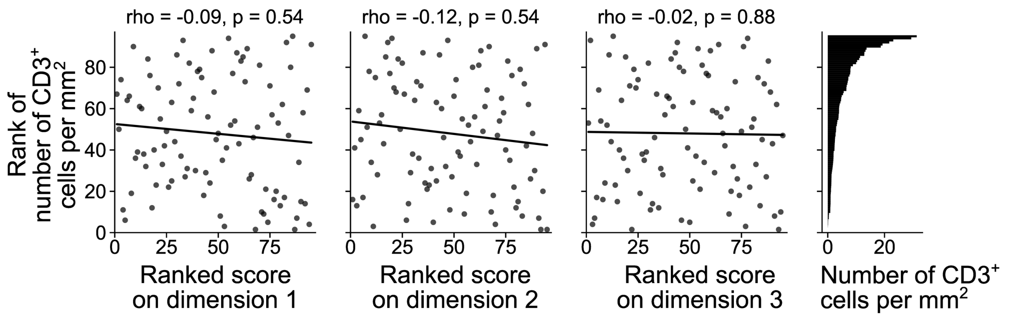

Suppl. Fig. 14 Association between $CD3^+$ cells per mm^2 in the normal-appearing white matter of the pyramidal tract and dimensions. Scatter plots with regression lines depict the rank of the number $CD3^+$ cells per mm^2 for 95 donors on the Y-axis, with donors ranked according to their score on dimension 1-3 on the X-axes. The bar plot depicts the unranked value on the X-axis, per rank on the Y-axis. Significance was assessed with Spearman correlation and FDR-adjusted for multiple testing. Ties were assigned averaged ranks. FDR = False Discovery Rate

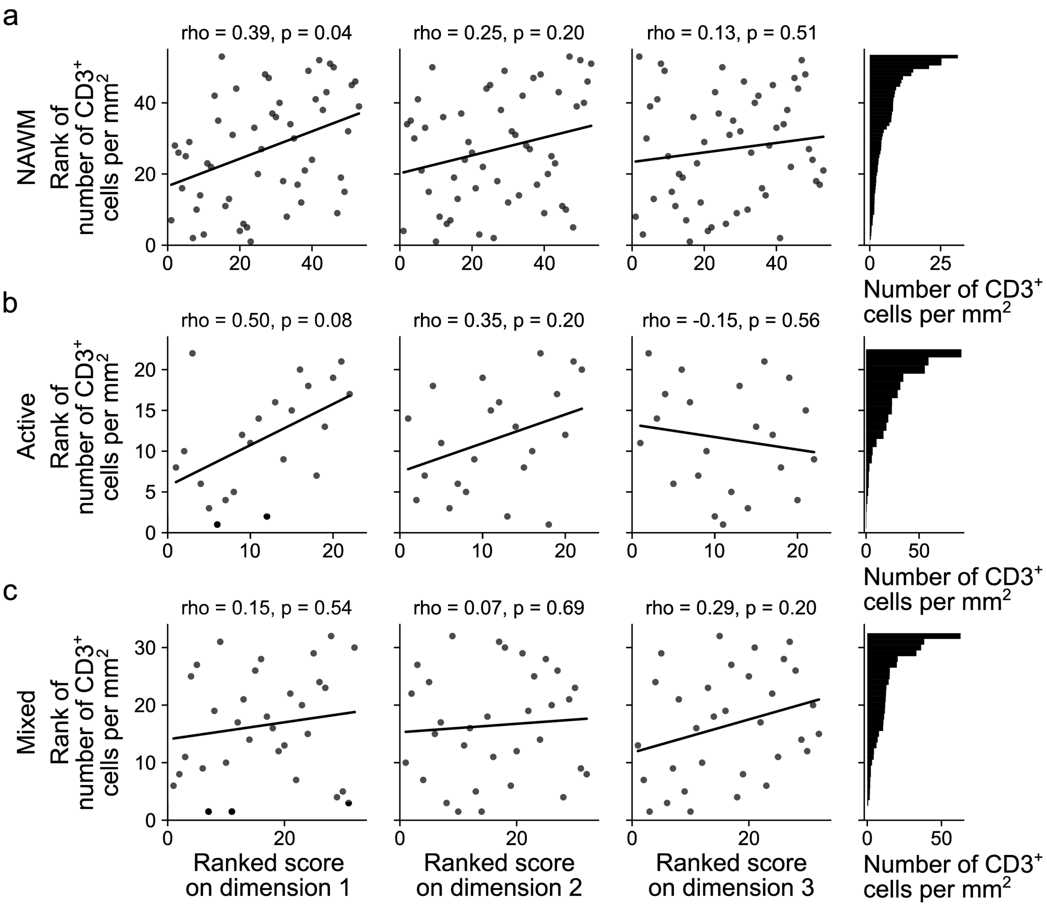

Suppl. Fig. 15 Association between subcortical CD3$^+$ cells per mm^2 and dimensions. **a-c** Scatter plots with regression lines, depicting on the Y-axis the rank of the number of CD3$^+$ cells per mm^2 in subcortical normal-appearing white matter (NAWM) for 53 donors (**a**), in subcortical active lesions for 22 donors (**b**), and in subcortical mixed lesions for 32 donors (**c**), with donors ranked according to their score on dimension 1-3 on the X-axes. The bar plots depict the unranked value on the X-axis, per rank on the Y-axis. Significance was assessed with Spearman correlation and FDR-adjusted for multiple testing. Ties in **a-c** were assigned averaged ranks. FDR = False Discovery Rate

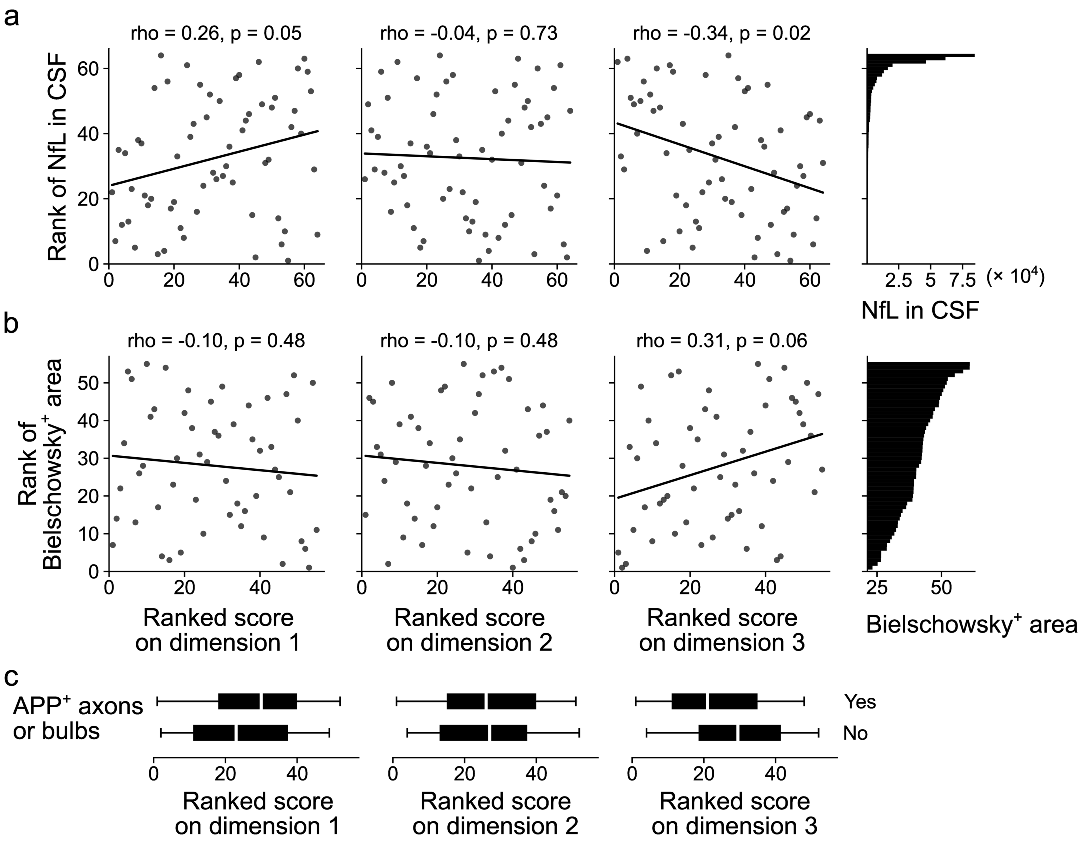

Suppl. Fig. 16 Association between neuroaxonal damage and dimensions. **a-b** Scatter plots with regression lines, depicting on the Y-axis the rank of neurofilament light chain (NfL) levels in cerebrospinal fluid (CSF) for 64 donors (**a**), and the rank of axonal density, quantified as the percentage of Bielschowsky positive area in the normal-appearing white matter of the pyramid tract, for 55 donors (**b**), with donors ranked according to their score on dimension 1-3 on the X-axes. The bar plots depict the unranked value on the X-axis, per rank on the Y-axis. Significance was assessed with Spearman correlation and FDR-adjusted for multiple testing (separately for NfL levels and axonal density). **c** Box plots showing the ranked scores of donors with and without amyloid precursor protein (APP) positive axonal fragments or bulbs, per dimension. Presence or absence of APP^+ fragments or bulbs was known for 52 donors (25 with, 27 without). There were no significant differences in scores on dimensions (Mann-Whitney U; FDR-adjusted p-values: dim. 1: $p = 0.43$; 2: $p = 0.99$; 3: $p = 0.42$). Ties in **a-c** were assigned averaged ranks. FDR = False Discovery Rate

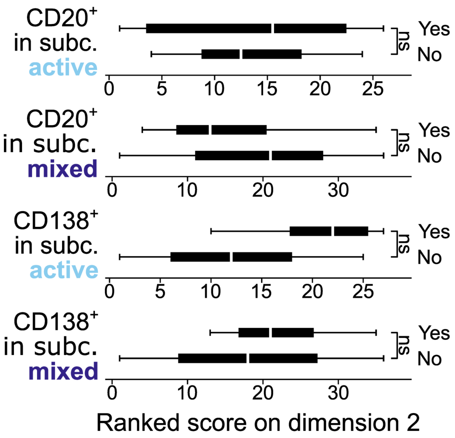

Suppl. Fig. 17 Association between B cells in subcortical (subc.) lesions and dimension 2. Box plots showing the ranked scores of 26 donors with (10) and without (16) $CD20^+$ cells in subcortical active lesions, of 36 donors with (11) and without (25) $CD20^+$ cells in subcortical mixed lesions, of 27 donors with (6) and without (21) $CD138^+$ cells in subcortical active lesions, and for 36 donors with (4) and without (32) $CD138^+$ cells in subcortical mixed lesions, from top to bottom respectively. Ties were assigned averaged ranks. Significance was assessed with Mann-Whitney U and FDR-adjusted for multiple testing (for the comparison of scores on dimension 1-3 for donors with and without $CD20^+$ cells in subcortical active and mixed lesions, and separately for the comparison of scores on dimension 1-3 for donors with and without $CD138^+$ cells in subcortical active and mixed lesions). ns $p > 0.1$; + $p \leq 0.1$; * $p \leq 0.05$; ** $p \leq 0.01$; *** $p \leq 0.001$; **** $p \leq 0.0001$; FDR = False Discovery Rate

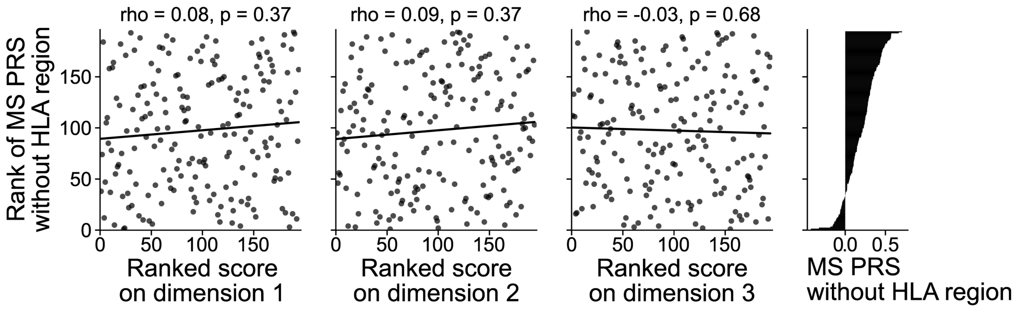

Suppl. Fig. 18 Association between dimensions and non-HLA polygenic risk score (PRS), calculated by excluding the extended HLA region on chromosome 6 (base pairs 24,000,000 to 35,000,000; genome assembly GRCh37/hg19). Scatter plots with regression lines depict the rank of the PRS without HLA region for 194 donors on the Y-axis, with donors ranked according to their score on dimension 1-3 on the X-axes. The bar plot depicts the unranked value on the X-axis, per rank on the Y-axis. Significance was assessed with Spearman correlation and FDR-adjusted for multiple testing. Ties were assigned averaged ranks. HLA = human leukocyte antigen; FDR = False Discovery Rate
